# Digital phenotyping of affect and stress in emerging adults

**DOI:** 10.3389/fdgth.2026.1799541

**Published:** 2026-06-04

**Authors:** Coralie S. Phanord, Luka L. Ruzic, Siddharth Kalyanasundaram, Sofia Barnes-Horowitz, Naomi P. Friedman, Theodora Chaspari, Roselinde H. Kaiser

**Affiliations:** 1 Department of Psychology and Neuroscience, University of Colorado Boulder, Boulder, CO, United States; 2 Department of Computer Science, University of Colorado Boulder, Boulder, CO, United States; 3 Institute for Behavioral Genetics, University of Colorado Boulder, Boulder, CO, United States; 4 Institute of Cognitive Science, University of Colorado Boulder, Boulder, CO, United States; 5 Renée Crown Wellness Institute, University of Colorado Boulder, Boulder, CO, United States

**Keywords:** affect, digital phenotyping, ecological momentary assessment, machine learning, mood, passive sensing, smartphone, stress

## Abstract

**Background:**

Depression is highly heterogeneous and difficult to monitor or predict in daily life. One strategy for monitoring depressive symptoms is digital phenotyping, the real-time tracking of behaviors via personal devices. Digital phenotyping may be especially useful for predicting mood in emerging adults, a developmental period characterized by heightened rates of depression and smartphone use. However, prior research lacks long-term data and rigorous comparison of modeling approaches.

**Objective:**

The present pre-registered study addresses these gaps by modeling smartphone sensor-based behavioral markers to predict affect and stress over a one-year period in emerging adults (*n* = 24, ages 18–21), and comparing competing modeling approaches.

**Methods:**

Measures included daily ecological momentary assessment and continuous collection of sensor data from smartphones. Behaviors were estimated as features reflecting sleep, activity, mobility, and phone use, and tested as predictors of daily affect and stress in supervised machine learning models. Comparisons were performed across idiographic and nomothetic XGBoost models: Model 1 Group General that estimated behaviors that predict affect/stress across people, Model 2 Group Personalized that allowed for person-level estimation of behaviors that predict affect/stress, and Model 3 Within-Person Personalized which fit models independently for each participant.

**Results:**

Models provided complementary insights and showed different strengths and weaknesses. Model 1 Group General identified behaviors that predicted daily affect/stress across people, but showed poor generalizability. The personalized models (Model 2–3) outperformed general models, and Model 2 Group Personalized offered the best balance of accuracy and stability across evaluation metrics. However, participant ID contributed most of that model's predictive power, suggesting that the model primarily captured stable individual differences in affect/stress. In contrast, Model 3 Within-Person Personalized revealed person-specific patterns of behaviors predicted daily affect, but model reliability was limited.

**Conclusions:**

Findings reveal complementary strengths and weaknesses from machine learning models spanning the idiographic–nomothetic spectrum, for predicting affect and stress from passively sensed behavioral features. These results highlight the need for future research to rigorously compare, and quantify strengths and weaknesses, of personalized and hybrid modeling strategies that predict affective and stress outcomes. Insights from this study can guide future digital phenotyping research, which is crucial for exploring translational applications.

## 1 Introduction

Depression imposes a significant burden in terms of cost, disability, morbidity, and mortality (1–3). Depression is also highly prevalent (4–6), particularly among emerging adults, ages 18–25, who have the highest prevalence rate among all age groups (17.2%) (7). Emerging adulthood is a developmental period marked by life transitions and increased psychiatric vulnerabilities, which may be due to continued brain development, increasing autonomy, and identity formation (8, 9). Therefore, monitoring risk factors and fluctuations in depressive symptoms in emerging adults is crucial for identifying at-risk individuals and facilitating early or just-in-time interventions.

### 1.1 Monitoring affect and stress

Depression is highly heterogeneous (10) and difficult to monitor or predict in daily life (11, 12). Traditional monitoring methods rely on retrospective self-reports collected during infrequent clinical visits, which are often time-consuming and expensive (12, 13). Retrospective self-report measures typically ask about perceived mood over limited recent periods of time, which can be subject to temporal bias (14). Retrospective self-reports also risk patient recall bias, decreased illness insight, and individual observer bias, potentially misrepresenting daily experiences (12, 14, 15).

Digital phenotyping offers an alternative solution for monitoring depression. Digital phenotyping, defined by Torous and colleagues (16) as the “moment-by-moment quantification of the individual-level human phenotype *in-situ* using data from smartphones and other personal digital devices,” allows for remote, in-the-moment monitoring of behavior and emotions. This approach is promising for predicting or monitoring real-world depressive symptoms (11, 12, 17), e.g., disturbances in affect, and related experiences, e.g., subjective stress. Digital phenotyping can be achieved with data collected passively (e.g., smartphone sensor) or actively (e.g., survey) (16). Passive and active sensing methods are particularly suited for emerging adults, who have higher smartphone ownership than other groups (18).

Active data collection relies on user input (e.g., surveys) (16). For example, active data collection includes ecological momentary assessments (EMA), which are methods used to repeatedly gather self-reported information on behaviors, experiences, and context as they occur in natural settings (19). EMAs are increasingly being used to study psychological processes (20) through self-reporting and frequent surveys. Unlike retrospective assessments typically used in clinical settings, EMA captures affect in real-time, minimizing recall bias and improving ecological validity. However, individuals with depressive symptoms may be less likely to engage with EMA tools over long durations due to behavioral changes like demotivation and apathy (21). Further, in emerging adults, long-term adherence to EMA has been shown to decrease over time (22). Challenges in adherence to high-density EMA emphasize the importance of developing alternative or complementary mood monitoring strategies that do not exclusively rely on active user input.

Passively collected data is generated without active user participation (e.g., smartphone global positioning system (GPS) or accelerometry). The combination of active (EMA) and passive sensing provides a flexible framework: EMA data can be used to train models that, over time, rely more heavily on passive inputs. This approach supports scalable and low-burden monitoring, with the potential to identify changes in affect or stress with minimal user input. Smartphones and wearable devices are already integrated into the daily lives of emerging adults (23, 24) and research has shown that the sensors in these devices can unobtrusively predict changes in mood (25–29). Further, over the past decade, there has been a growing research focus on using smartphone log (30, 31), GPS (31, 32), and accelerometer data (33, 34) to extract behavioral features for predicting emotional states.

### 1.2 Evidence for behavioral markers of affect from personal sensing

A growing body of work demonstrates that smartphone sensors can passively capture behavioral patterns associated with depression (17). These include reduced mobility, lower physical activity, disrupted circadian rhythms, sleep disturbances, and distinct phone use behaviors, each of which has been studied as a potential digital correlate of mood or affective state (35–40). For example, depression appears to be characterized by reduced behavioral activation, a core concept in behavioral activation theory, which posits that decreased engagement in goal-directed or rewarding activities contributes to the maintenance of depressive symptoms (41). In line with behavioral activation theory, individuals with depression often exhibit reduced physical activity (42, 43), which can be captured using smartphone accelerometers (37, 44), as well as lower mobility, as shown by reduced GPS-based location variance (25, 38). Disruptions in circadian rhythm and diurnal activity cycles, also observed in depression (45, 46), have been inferred through passive sensing of phone use behaviors such as temporal usage patterns and screen interaction frequency (35). Sleep disturbances, a symptom of depression (47), have also been estimated via smartphone log data, associated with mood and stress levels, with shorter sleep predicting lower mood and higher stress (39, 48, 49). Furthermore, phone use behaviors such as screen unlock events and active screen time have been associated with depression, impulsivity, and poor self-regulation (39, 40, 50–52). These behavioral features have also been identified as possible predictors of EMA affect (48, 53–55) and stress (39, 49, 55–59). Additionally, digital markers of behavioral variability have frequently been associated with depression (e.g., variability in activity, smartphone use, and sleep patterns) (17). These variability-based features quantify behavioral fluctuations, which may reflect routine disruptions associated with depression (60). Together, this work highlights the potential of digital behavioral markers as indicators of affect and related experiences (e.g., stress) in daily life.

### 1.3 Gaps

Prior research in digital phenotyping has made significant progress in identifying behavioral markers of mood and stress using both passive and active data. However, there are several gaps in prior research.

First, previous studies have typically constructed models from data collected for short time periods, such as weeks or months (61), which may fail to capture symptom fluctuations on longer timescales.

Second, modeling approaches vary in the extent to which they account for heterogeneity in digital risk markers across people. For example, prior studies using passively sensed features to predict psychological self-reports have typically employed general group-level or nomothetic modeling approaches (62). However, nomothetic models risk incorrectly drawing conclusions about the individual based on group-level characteristics (63). This idea was supported by Fisher and colleagues, who used intensive repeated-measures data to show that individual variance around group means was considerably larger within individuals than within groups (64). Further, prior research suggests that individuals may vary in how they interact with their smartphones (24) and in how associations between passively sensed data and psychological self-reports differ across individuals (59, 65).

While some studies have demonstrated that idiographic models can predict mood and stress more accurately than nomothetic models (59, 62, 66), rigorous, systematic comparisons between personalized and general approaches, particularly for daily mood prediction over extended data collection periods, remain relatively scarce. Moreover, few studies have evaluated personalized modeling strategies that place substantial weight on the individual while still leveraging information from group data (67). Together, these gaps highlight the need for research that compares idiographic and nomothetic modeling approaches for affect and stress prediction in real-world, long-term datasets.

### 1.4 Objectives

In this study, we collected passively sensed smartphone data from emerging adults for a period of up to one year, together with daily EMA of affective symptoms and stress. Data were processed to extract behavioral features from sensor data, and for quality assurance.

The overall goal was to compare supervised machine learning models spanning the idiographic–nomothetic spectrum to predict affect and stress. Model 1 Group General predicts affect and stress from behavioral features across people, Model 2 Group Personalized estimates behavioral features across people and allows for person-level estimation of features to predict affect and stress, and Model 3 Within-Person Personalized predicts affect and stress independently for each participant. Our first aim was to identify whether personalized models (Model 2–3) outperform general models (Model 1). Our second aim was to identify which behavioral features related to phone use, sleep, and activity contributed most strongly to predicting daily affect and stress. We hypothesized that activity, sleep duration and circadian routines, and phone usage would be predictive of daily affect, such that measures of (1) increased behavioral activation predicted higher happiness, lower sadness, and lower anhedonia, (2) lower sleep duration and circadian routine predicted increased stress, lower happiness, and higher sadness, (3) lower activity entropy predicted lower happiness, higher sadness, and increased anhedonia, (4) and increased phone usage would be predictive of stress and affect in a person-specific manner. Finally, our third aim was to identify whether there were features that were consistently predictive of affect among subgroups.

The study was pre-registered on the Open Science Framework (68) (https://osf.io/sjcgq) after data collection, but prior to analysis.

## 2 Methods

### 2.1 Participants

Participants were undergraduate students recruited at the University of Colorado Boulder from a parent study with independent aims and research procedures. Inclusion criteria required participants to be between 18 and 25 years old and to own a smartphone. Diagnostic exclusion criteria included a history of psychotic disorders, severe substance use disorders, or eating disorders. A total of 28 emerging adults were enrolled, including both iPhone and Android users.

For these analyses, 3 participants who used Android devices were excluded due to differences in data structure across operating systems, which introduced additional complexity in preprocessing and feature extraction. After preprocessing, one additional participant was excluded for having insufficient EMA samples (*s* = 4) for reliable cross-validation. This resulted in a final analytic sample of 24 participants ages 18–21 years (Table 1). Sample demographics can be found in Table 1.

**Table 1:** Demographic characteristics, *N* = 24.

Characteristic	Participants
Age (years), mean (SD)	19.08 (0.91)
Age (years), min—max (median)	18–21 (19)
Sex, *n* (%)	
Female	8 (66.7)
Male	16 (33.3)
Gender, *n* (%)	
Woman	8 (66.7)
Man	16 (33.3)
Transgender	0 (0.0)
Non-binary/Third gender/Two-spirit	0 (0.0)
Fluid/Dynamic	0 (0.0)
Prefer not to say	0 (0.0)
Race, *n* (%)	
White	22 (92.7)
Black or African American	0 (0.0)
American Indian or Alaska Native	0 (0.0)
Asian	1 (4.2)
Native Hawaiian or Pacific Islander	0 (0.0)
More than one race	1 (4.2)
Hispanic or Latino/a/x, *n* (%)	
Yes	3 (12.5)
No	21 (87.5)
Family Household Income, *n* (%)	
Less than $10,000	2 (8.3)
$10,000–$25,000	2 (8.3)
$25,000–$50,000	3 (12.5)
$50,000–$75,000	1 (4.2)
$75,000–$100,000	3 (12.5)
More than $100,000	13 (54.2)
Highest Parent Education, *n* (%)	
Less than high school	0 (0.0)
High school or GED	2 (8.3)
Some college/2-year degree	2 (8.3)
Bachelor's degree	6 (25.0)
Graduate/ professional degree	14 (58.3)
No applicable guardian	0 (0.0)

min, minimum; max, maximum; SD, standard deviation.

### 2.2 Procedure

For the parent study, participants completed a comprehensive baseline session, involving a psychiatric diagnostic evaluation, cognitive tasks, and a neuroimaging scan. At the baseline session, prospective participants were invited to join this one-year-long mobile sensing study. Additionally, at the one-year mark, participants completed a second diagnostic interview for the parent study. Other independent hypotheses using other data types collected in the parent study will be tested and reported elsewhere.

At enrollment, participants installed the Beiwe smartphone application (16, 69–71) under the guidance of research staff who ensured activation of necessary location services. Beiwe facilitates both passive sensing and active data collection, and has demonstrated effectiveness in capturing longitudinal, high-density behavioral data in clinical populations (16, 69).

Over the one-year study period, participants completed a brief daily EMA microsurvey, reporting in-the-moment affect and stress intensity. Smartphone sensor data were collected continuously each day. The Beiwe application delivered the microsurvey between 2:00 p.m. and 6:00 p.m. daily, and the survey expired after 24 h.

### 2.3 Measures

Demographic information was collected, including age, sex, gender, and ethnicity.

#### 2.3.1 Ecological momentary assessment

Participants completed a once-daily Ecological Momentary Assessment (EMA), delivered via the Beiwe application. To support compliance and minimize participant burden, EMA were designed to be short with a single-item format. EMA items used in this study measured affect and stress. These items include self-reported feelings of sadness, pleasure and interest, happiness, irritability or anger, and stress, each rated on a Likert scale ranging from 0 to 100. Each microsurvey required less than one minute to complete. Each participant completed a maximum of 365 EMA.

#### 2.3.2 Digital sensors

Sensor data were collected throughout each day. Sensor data included GPS coordinates, accelerometer readings, and phone log data. GPS and accelerometer data were collected for one minute at 10-minute intervals (one minute of data collection every 11 min). During the one-minute collection period, accelerometer data were gathered at a rate of 10 samples per second and GPS data at one sample per second. Phone log data collection was triggered by activity logged on the device.

### 2.4 Data processing

#### 2.4.1 Quality assurance

Processing of sensor and EMA data was performed to address missing data and outliers. The data passed through a quality assurance pipeline that only estimated features on days with completed EMA data. To ensure a sufficient period of data coverage for each specific sensor (e.g., GPS or accelerometer) over the course of a day, we applied a threshold of 10 h based on data coverage across the sample. We also discarded days on which there were periods of 8+ hours without sensor data collected. For phone log data, we imputed a minimum phone use duration for each phone unlock event that was not paired with a lock event. However, we discarded unlock events that were not paired with a lock event within a period of one hour. We also filtered out feature outliers, defined as measurements above +4 SD. Further, we discarded days between Sept 10th 2022 – Jan 29th 2023, *a priori* due to server misbehavior. Prior to preprocessing, the dataset contained 8,218 data points; after preprocessing, 2,786 data points remained.

To examine the impact of user data availability on model performance and model predictions, we conducted Spearman correlation analyses between the amount of user data and two outcomes: (1) model predictions (mean EMA scores) and (2) model performance metrics (RMSE and R^2^) measured by fold and by dataset. Additionally, to assess whether EMA missingness was associated with passively sensed behavioral patterns, we fit a series of generalized linear mixed models for each z-normalized behavioral feature, with a random intercept accounting for participant repeated measures. Analyses related to quality assurance were performed using R Statistical Software (v4.4.2; R Core Team 2024). Results from correlation analyses and generalized linear mixed models are reported in the Supplementary Material.

#### 2.4.2 Feature extraction

We extracted 34 behavioral features. To reduce collinearity among features, we grouped features with strong positive correlations (*r* ≥ 0.6) into composite variables, so that each composite reflected a coherent underlying construct, resulting in 21 total features (Table 2). A matrix of the pairwise correlations is provided in the Supplementary Material. Our in-house feature extraction captured daily summary statistics from GPS, accelerometer, and phone log data. These summary statistics included minutes of phone use, total phone unlocks, distance traveled, activity space, and daily median, maximum, and standard deviation of acceleration. Additionally, we utilized the Reproducible Analysis Pipeline for Data Streams (RAPIDS) pipeline (72), an open-source platform that supports feature extraction from data collected via mobile devices. We incorporated all features in RAPIDS Phone Location to estimate 14 GPS-based mobility metrics. Notably, among these metrics, Circadian Routine and Weekend Circadian Routine were highly correlated with Home Time. Therefore, they were interpreted as indices of the duration and regularity of time spent at home, and were combined to create the composite feature Home Location Regularity. For sleep duration estimation, we employed an in-house rule-based algorithm based on Aalbers et al. (59), which utilizes phone log data (from screen unlocks) to identify sleep start and end times. This method, initially designed for screen unlock data, was also adapted for accelerometer-based and GPS-based stationary data. To account for missing data, sleep duration was calculated as the overlapping period between the sleep duration estimates for screen unlocks and stationary status.

**Table 2:** Behavioral features.

Features	Sensor
Unlocks* [Unlocks, Unlocks Day]	Phone log
Unlocked Mins* [Unlocked Mins, Unlocked Mins Day]	Phone log
Unlocks Night	Phone log
Unlocked Mins Night	Phone log
Travel* [Distance *(travelled),* Span, Convex Area, Rog, Max Diam]	GPS
Flight Length* [Ave Flight Length, SD Flight Len]	GPS
Sig Loc* [Sig Loc Visited, Sig Loc Entropy]	GPS
Buffer Area *(buffer activity space)*	GPS
Pause *(the fraction of the day spent in a pause)*	GPS
Max Dist Home *(maximum distance from home)*	GPS
Ave Flight Dur	GPS
SD Flight Dur	GPS
Home Loc Reg* [Circadian Routine, Weekend Circadian Routine, Home Time]	GPS
Mdn Acc* [Mdn Acc, Mdn Acc Day]	Accelerometer
SD Acc* [SD Acc, SD Acc Day]	Accelerometer
Max Acc Night	Accelerometer
Mdn Acc Night	Accelerometer
SD Acc Night	Accelerometer
Max Acc	Accelerometer
Max Acc Day	Accelerometer
Sleep Dur	All

*, composite; Mins, minutes; Max, maximum; SD, standard deviation; Mdn, median; Acc, magnitude of acceleration; Ave, average; Dur, duration; Sig, significant; Loc, location(s); Reg, regularity; Diam, diameter; rog, radius of gyration.

### 2.5 Analyses

Primary analyses were performed in Python (version 3.8.18) with the scikit-learn library (73).

We developed idiographic and nomothetic supervised machine learning models to identify passively sensed behavioral features that predicted self-reported affect and stress across the group or for individual participants (see model types below). We specifically utilized eXtreme Gradient Boosting (XGBoost) regression, which has been successfully used in previous work (67, 74). We selected XGBoost over other machine learning models since they offer interpretable, hierarchical decision structures that can capture non-linear relationships between input features and outputs, while maintaining low computational cost during both training and testing. For analyses designed to isolate within-person variance on a comparable scale across participants, we normalized each participant's EMA microsurvey responses using *Z*-score normalization around their respective means and standard deviations.

Additionally, we assessed whether personalized models outperformed general models by implementing three XGBoost regression approaches. (1) Model 1 Group General was trained across all participants without including participant identity. (2) Model 2 Group Personalized was trained across all participants and included participant ID as a feature. By including participant ID, the model split decision paths based on person-specific patterns, effectively tailoring predictions to individuals within a group-level structure. (3) Model 3 Within-Person Personalized models were trained separately on each individual's data.

Modeling was performed using k-fold cross-validation for a more reliable estimate of performance. To optimize model parameters, hyperparameter tuning was conducted on the cross-validation train sets. Details on cross-validation and tested hyperparameter values are reported in the Supplementary Material. We quantified feature importance using the pre-registered permutation importance method and evaluated model performance using Root Mean Squared Error (RMSE) and R-squared (R^2^). R^2^ reflects the proportion of variance explained by the model, with higher values indicating a better fit and negative values indicating performance worse than simply predicting the mean. RMSE captures the average prediction error, with lower values indicating a better fit. Because RMSE scales with the variance of each outcome, it should be compared within the same item. In addition to the regression models, we also conducted classifier versions of these analyses for comparison, as preregistered; results of the classification models are reported in the Supplementary Material.

As exploratory analyses, we used SHAP (SHapley Additive exPlanations) to examine the direction and magnitude of feature contributions and conducted intercept-only model comparisons to evaluate the added predictive value of our models. Further, we compared the XGBoost Model 2 Personalized Group model, which incorporated both participant ID and behavioral features, against three comparison models: (1) Model 1 (the Group General model), meaning only behavioral features were included, (2) a model including only participant ID, and (3) an intercept-only model. Additionally, to explore data-driven subgroups of emerging adults with distinctive profiles of depression risk, we conducted a k-means clustering analysis on the sad/down Model 2 Group Personalized. K-means clustering analysis results are reported in the Supplementary Material.

## 3 Results

### 3.1 Sample characteristics

**Figure 1:**
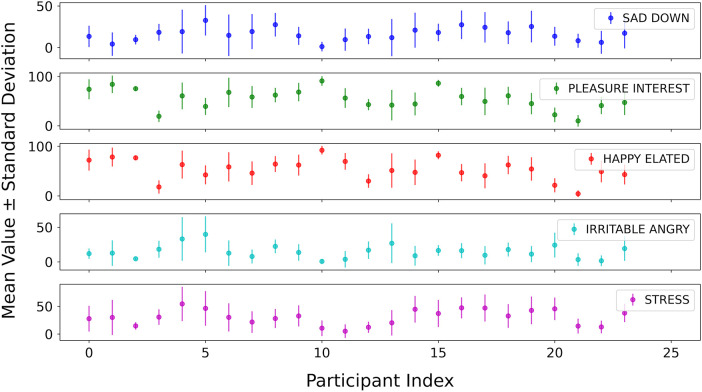
Distribution of daily affect ratings (EMA items).

Descriptive statistics for daily affect ratings can be found in Table 3 and Figure 1.

**Table 3:** Daily affect rating distribution.

EMA Item	Mean (SD)
Sad Down	16.16 (18.38)
Pleasure Interest	54.24 (27.91)
Happy Elated	53.77 (28.76)
Irritable Angry	14.12 (18.75)
Stress	30.16 (25.33)

EMA, ecological momentary assessment; SD, standard deviation.

### 3.2 Model performance

**Figure 2:**
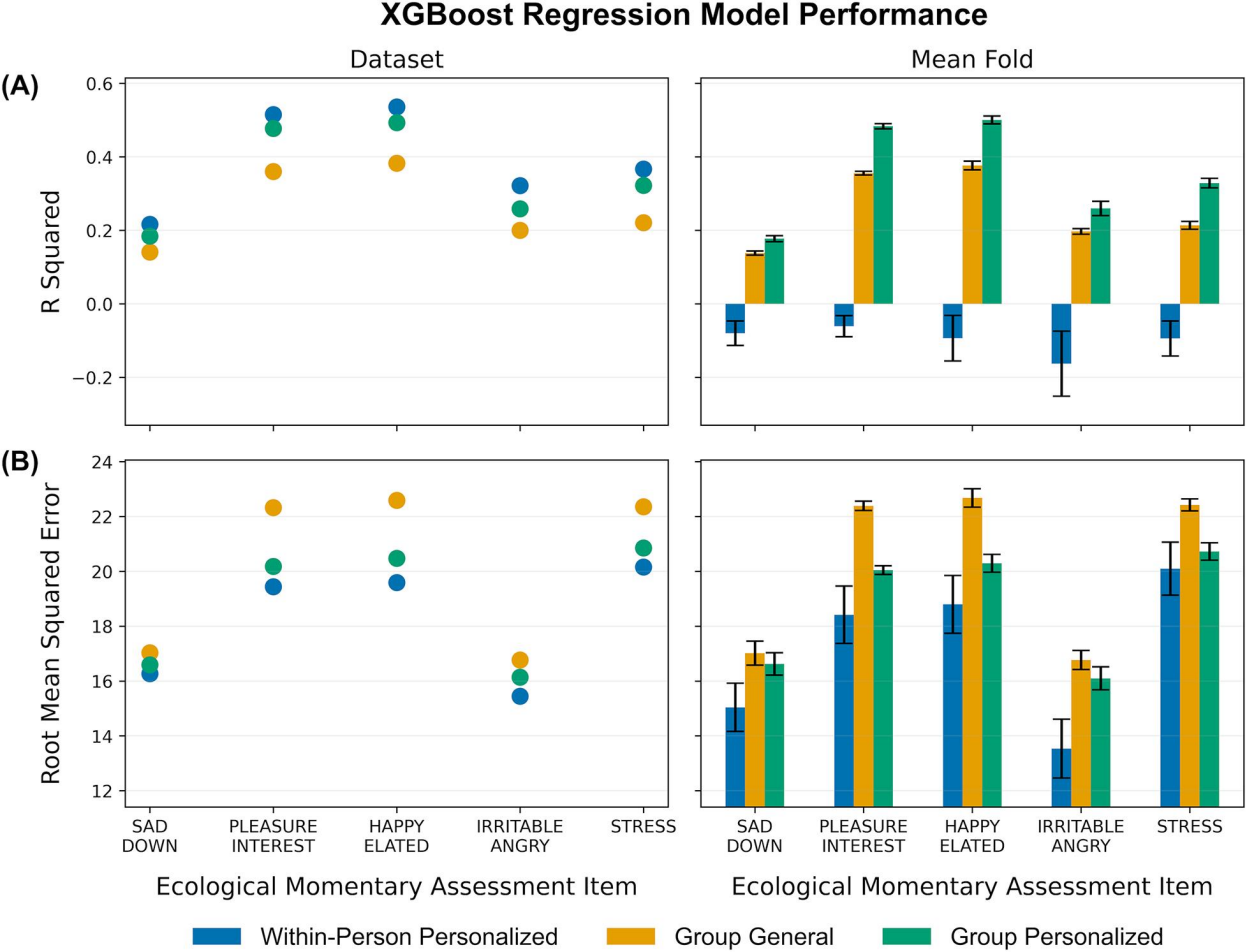
XGBoost regression performance metrics. Plots on the left display dataset-level metrics, meaning metrics were calculated by aggregating predictions across all data points, rather than averaging across individual cross-validation folds. Each point represents the performance of a model using the full dataset. Plots on the right display metrics measured by fold, meaning performance metrics were averaged across cross-validation folds, with each fold's performance computed separately before aggregation. Bars represent the mean performance across folds, and error bars indicate standard error. The performance metrics displayed are **(A)** estimated R^2^, where higher values indicate better fit, and **(B)** estimated Root Mean Squared Error, where lower values indicate better fit.

Model 1 Group General models yielded RMSE ranging from 16.769 (*irritable/angry*) to 22.591 (*happy/elated*), and R^2^ of 0.141 (*sad/down*) to 0.383 (*happy/elated*) (Figure 2).

Model 2 Group Personalized models, which were trained across the group but included participant ID as a feature, yielded RMSE ranging from 16.141 (*irritable/angry*) to 20.852 (*stress*), and R^2^ of 0.184 (*sad/down*) to 0.493 (*happy/elated*) (Figure 2).

Model 3 Within-Person Personalized models, trained within-subject, yielded RMSE of 15.442 (*irritable/angry*) to 20.155 (*stress*), and R^2^ of 0.216 (*sad/down*) to 0.536 (*happy/elated*). However, we note that R^2^ values computed by pooling predicted values from all cross-validation sets (herein referred to as “fold-level”) were consistently negative for within-subject personalized models, ranging from −0.163 (*irritable/angry*) to −0.061 (*pleasure/interest*). A negative R^2^ indicates the model performed worse than simply predicting the mean. This discrepancy between dataset-level and fold-level metrics suggests that within-person sample sets may have been too small for reliable validation within individual participants, an observation that could explain the discrepancies in terms of R^2^ values between the dataset-level metrics and fold-level metrics (Figure 2).

Figure 2 and Table 4 provide an overview of how accurately Model types 1–3 predict out-of-sample data. Together, these results indicate that personalized models (Models 2, 3) generally outperformed the general models (Model 1) across all affective outcomes. While Model 3, within-subject personalized models achieved the lowest RMSE, their poor fold-level R^2^ performance limits their reliability. Model 2, by contrast, demonstrated the highest fold-level R^2^ values and consistently lower RMSE than other models, suggesting that they offer the best compromise between accuracy and stability.

**Table 4:** XGBoost regression performance metrics (mean across folds).

Model	EMA	RMSE (SE)^a^	R^2^ (SE)	Pearson's r
Group General	Sad Down	17.016 (0.438)	0.138 (0.006)	0.375
	Pleasure Interest	22.385 (0.170)	0.355 (0.005)	0.611
	Happy Elated	22.675 (0.333)	0.376 (0.012)	0.626
	Irritable Angry	16.765 (0.349)	0.197 (0.008)	0.447
	Stress	22.421 (0.216)	0.213 (0.011)	0.470
Group Personalized	Sad Down	16.622 (0.406)	0.177 (0.008)	0.429
	Pleasure Interest	20.040 (0.159)	0.483 (0.007)	0.693
	Happy Elated	20.291 (0.323)	0.500 (0.011)	0.703
	Irritable Angry	16.096 (0.418)	0.259 (0.019)	0.509
	Stress	20.722 (0.320)	0.328 (0.013)	0.568
Within-Person Personalized	Sad Down	15.041	−0.080	0.466
	Pleasure Interest	18.415	−0.061	0.732
	Happy Elated	18.795	−0.094	0.718
	Irritable Angry	13.535	−0.163	0.568
	Stress	20.095	−0.094	0.606

a Standard error is not included in Model 3 Within-Person Personalized models as metrics were computed from 2-fold cross validation for those models due to smaller sample size in individual models. We implemented 5-fold cross-validation for Models 2–3 and include standard error. See the Supplementary Material for more information on cross-validation folds for each model type.

RMSE, root mean square error; SE, standard error; Acc, acceleration; EMA, ecological momentary assessment; Pearson's r, Pearson's correlation measuring the linear association between predicted and actual values.

Classification models were also examined, as outlined in the preregistration. However, in contrast to the regression results, classification models exhibited limited differentiation between approaches, with relatively small variations observed in weighted F1-scores and balanced accuracy. Full results for classification analyses are presented in the Supplementary Material.

### 3.3 Comparison to baseline models

We compared the performance of Models 1–3 to an intercept-only model (a corresponding baseline using the same XGBoost model structure but with all features set to zero). Additionally, we compared Model 2 against Model 1 (only features as predictors), and against a model that only evaluated participant differences in affect (only participant ID as predictors).

**Figure 3:**
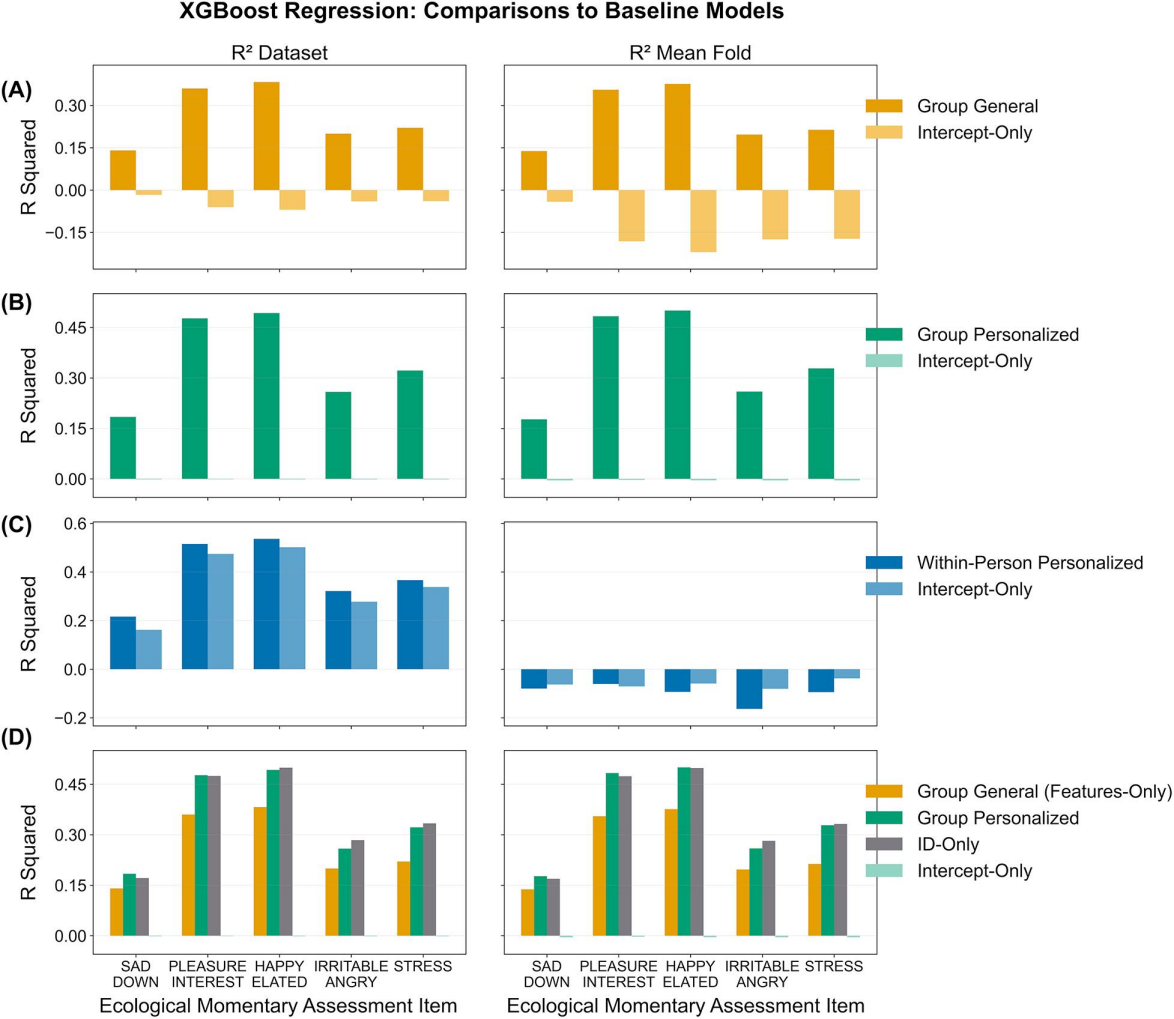
XGBoost regression comparisons to XGBoost baseline models **(A)** Bar plots comparing R^2^ for model 1 group general models and intercept-only models calculated by dataset and by fold. **(B)** Bar plots comparing R^2^ for Model 2 Group Personalized models and Intercept-only models calculated by dataset and by fold. **(C)** Bar plots comparing R^2^ for Model 3 Within-Person Personalized models and Intercept-only models calculated by dataset and by fold. **(D)** Bar plots comparing R^2^ Model 2 Group Personalized models (features and participant ID included in model) and ID-only models, Model 1 feature-only models (equivalent to Group General Models), and Intercept-only models calculated by dataset and by fold.

First, comparing Model 1 Group General models to intercept-only models, the intercept-only models achieved R^2^ values ranging from −0.069 to −0.017 when evaluated by dataset and from −0.219 to −0.041 when evaluated by fold (see Figure 3A). In comparison, the Model 1 Group General models achieved R^2^ values ranging from 0.141 to 0.383 by dataset, and from 0.138 to 0.376 by fold. These results demonstrate that Group General models captured meaningful variance in affective states across participants compared to the intercept-only baseline.

Second, comparing Model 2 Group Personalized models to intercept-only models, the intercept-only baseline achieved R^2^ values ranging from −0.002 to −0.001 (see Figure 3B). In contrast, Model 2 Group Personalized models showed R^2^ values ranging from 0.184 to 0.493 by dataset, and from 0.177 to 0.500 by fold.

Comparing Model 2 Group Personalized models to models that only included participant ID as the predictor (effectively modeling between-person differences in average affect or stress), the ID-only models achieved R^2^ values ranging from 0.171 to 0.499 by dataset and from 0.169 to 0.498 by fold (see Figure 3D). Hence, including behavioral features appeared to provide limited or no additional explanatory power in these models, over and above the participant's average affect or stress.

Finally, as shown in Figure 3D, Model 1 (feature-only Group General) performed better than intercept-only models, but not as well as Model 2 (models including participant ID).

Third, comparing Model 3 Within-Person Personalized models to intercept-only models, the intercept-only models achieved R^2^ values ranging from 0.162 to 0.501 when evaluated by dataset, and from −0.081 to −0.038 when evaluated by fold (see Figure 3A). In comparison, models including features achieved R^2^ values ranging from 0.22 to 0.54 by dataset, and from −0.163 to −0.061 by fold.

### 3.4 Cross-Validation comparisons

**Figure 4:**
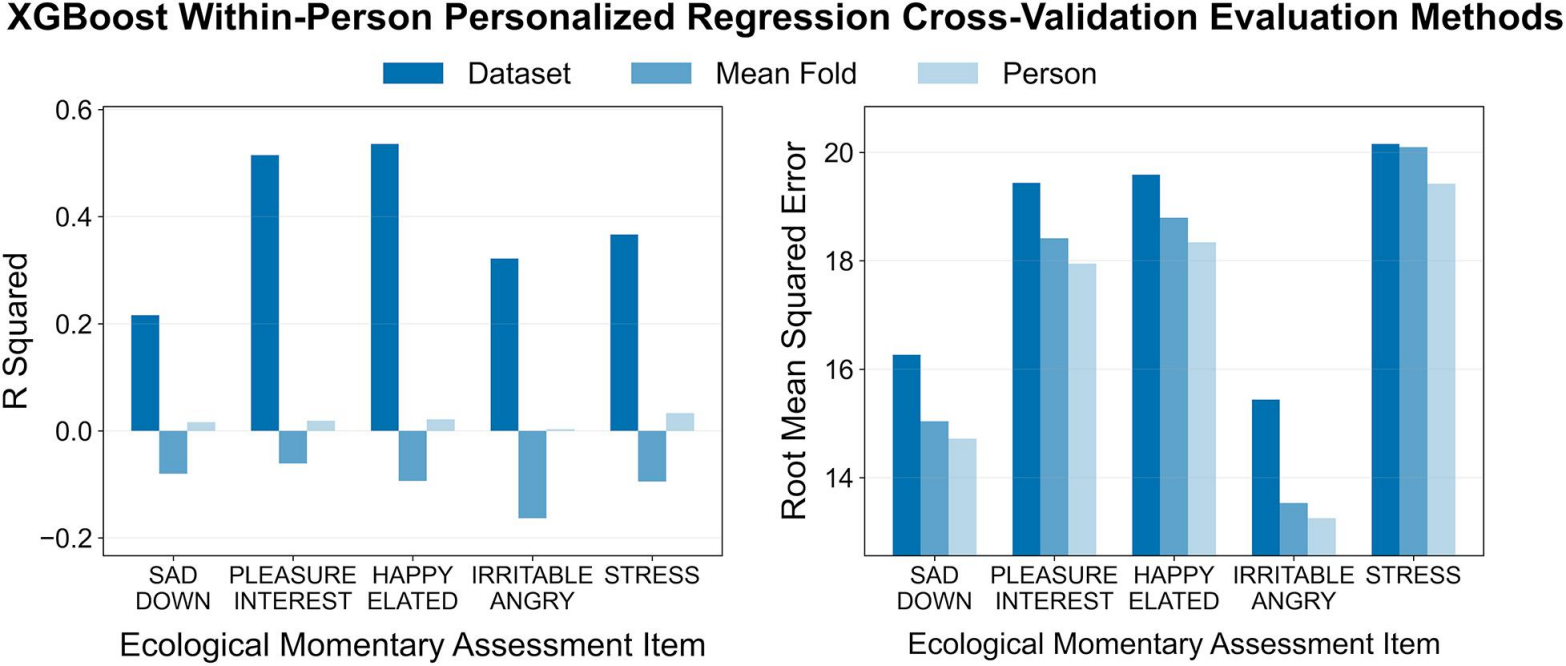
Bar plots comparing performance metrics (RMSE and R^2^) for model 3 within-person personalized models, measured on the dataset-level, meaning metrics were calculated by aggregating predictions across all data points, by fold, meaning performance metrics were averaged across cross-validation folds, and by subject, meaning performance metrics were averaged across subjects.

As noted above, for Model 3 Within-Person Personalized, we observed discrepancies in model performance metrics when calculated by pooling across all cross-validation sets vs. computing them at the dataset-level in within-subject personalized models. Figure 4 displays R^2^ and RMSE values computed using three approaches: (1) the mean of metrics across cross-validation folds, (2) the mean of per-participant metrics, and (3) metrics calculated from aggregated predictions across the entire dataset. R^2^ values varied substantially between these methods.

**Figure 5:**
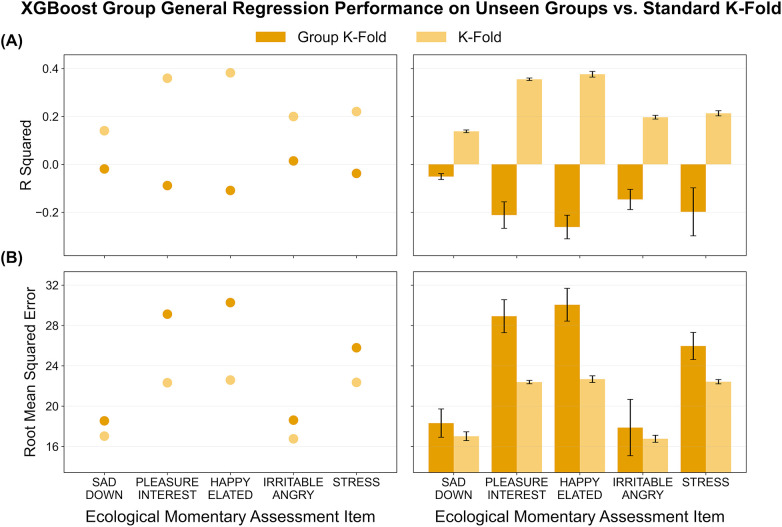
XGBoost model 1 group general regression performance metrics using group k-fold vs. standard k-fold. The performance metrics displayed are **(A)** estimated R^2^ and **(B)** estimated RMSE. Group K-Fold cross-validation preserves participant grouping by ensuring that all data from a single participant appears in only one fold, avoiding data leakage. In contrast, standard K-Fold splits the data randomly across folds, which may result in the same participant's data appearing in both training and validation sets. Plots on the left display dataset-level metrics, meaning metrics were calculated by aggregating predictions across all data points, rather than averaging across individual cross-validation folds. Each point represents the performance of a model using the full dataset. Plots on the right display metrics measured by fold, meaning performance metrics were averaged across cross-validation folds, with each fold's performance computed separately before aggregation. Bars represent the mean performance across folds, and error bars indicate standard error.

A second area of comparison was the performance of Model 1 Group General models with competing approaches for test/train datasets (see Figure 5). While the primary performance results (Figure 2) were based on standard k-fold cross-validation, we also compared this approach to grouped k-fold, in which participant groups were fully held out from the training set. Models performed poorly when using grouped k-fold, with R^2^ values ranging from −0.11 to 0.01, across different EMAs, indicating worse performance than simply predicting the mean.

### 3.5 Feature importance

#### 3.5.1 Model 1 group general

**Figure 6:**
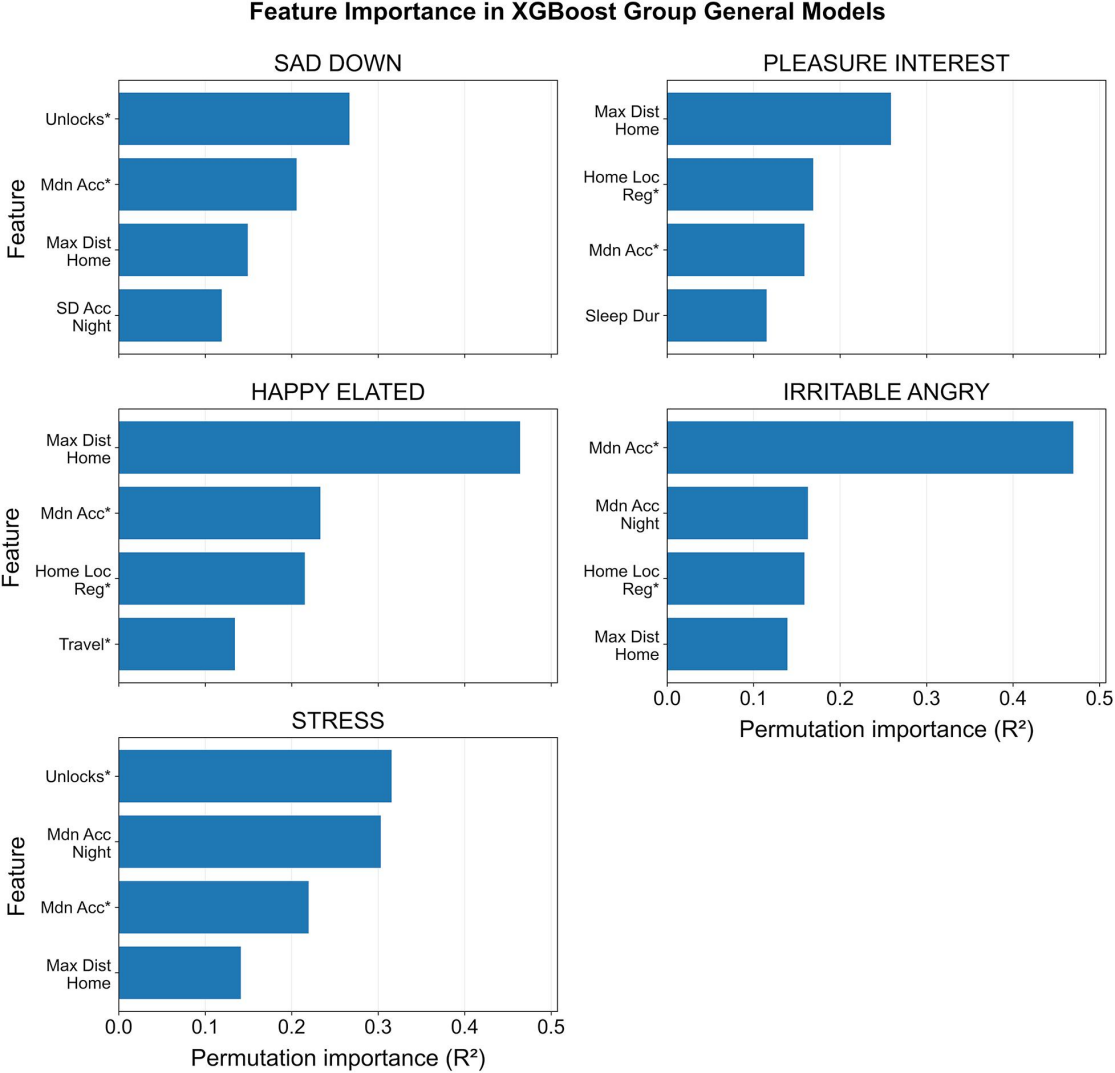
Bar plots of top four features based on the permutation importance method in model 1 group general models.

In feature importance analysis of Model 1 Group General, behavioral features reflecting activity, mobility, and phone usage showed the highest contributions to predictions. The permutation importance of the top four most important features, ranged from 0.12 to 0.27 for *sad/down* and *pleasure/interest*, 0.13 to 0.46 for *happy/elated*, 0.29 to 0.47 for *irritable/angry*, and 0.14 to 0.32 for stress. The features appearing most important were Phone Unlocks, Median Magnitude Acceleration (a potential proxy for physical activity), Location Regularity (a composite capturing GPS-based circadian regularity and time spent at home), and Maximum Distance From Home. See Figure 6 for the four most important features per EMA as derived from the permutation importance method (based on optimizing R^2^).

#### 3.5.2 Model 2 group personalized

**Figure 7:**
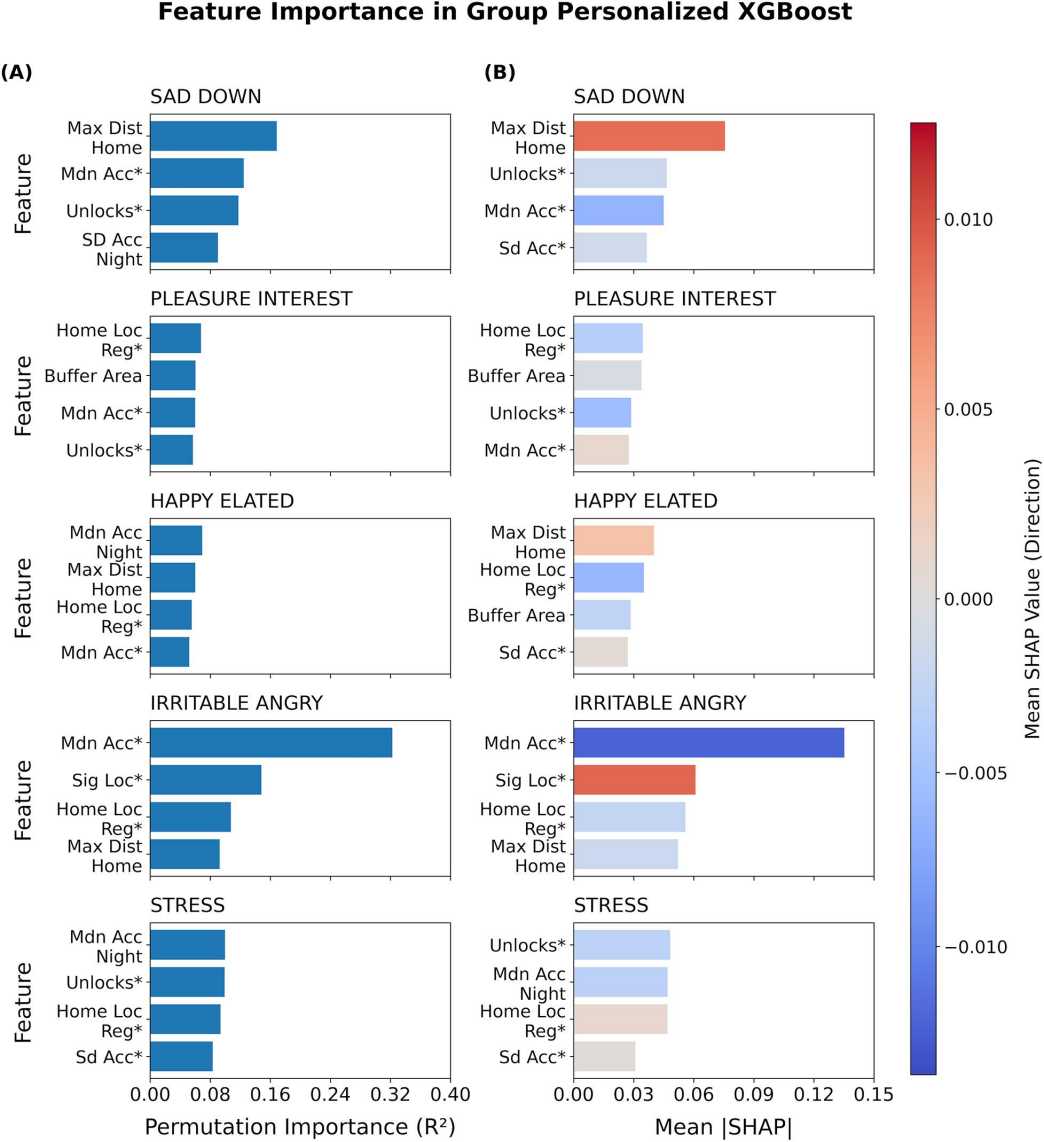
Bar plots of top four features in model 2 group personalized models, based on **(A)** the permutation importance method and **(B)** SHAP (SHapley additive exPlanations) in group personalized XGBoost models. Participant ID is not included.

In contrast, in Model 2 Group Personalized, behavioral features showed only modest contributions to prediction. The top four behavioral features by permutation importance ranged from 0.09 to 0.17 for *sad/down*, 0.06 to 0.07 for *pleasure/interest*, 0.05 to 0.07 for *happy/elated*, 0.09 to 0.32 for *irritable/angry*, and 0.08 to 0.10 for *stress*. Additionally, while mean absolute SHAP values were similarly modest, mean SHAP values across features and EMA models were near zero, ranging from −0.006 to 0.009 for *sad/down*, −0.001 to 0.003 for *pleasure/interest*, −0.006 to 0.003 for *happy/elated*, −0.012 to 0.01 for *irritable/angry*, and −0.004 to 0.008 for stress. Figure 7 displays the distribution of the four most important behavioral features per EMA as derived from the permutation importance method and SHAP. Notably, participant ID emerged as the most important feature across all EMA models, with permutation importance ranging from 0.39 (*sad/down*) to 0.97 (*happy/elated*).

#### 3.5.3 Model 3 within-person personalized

**Figure 8:**
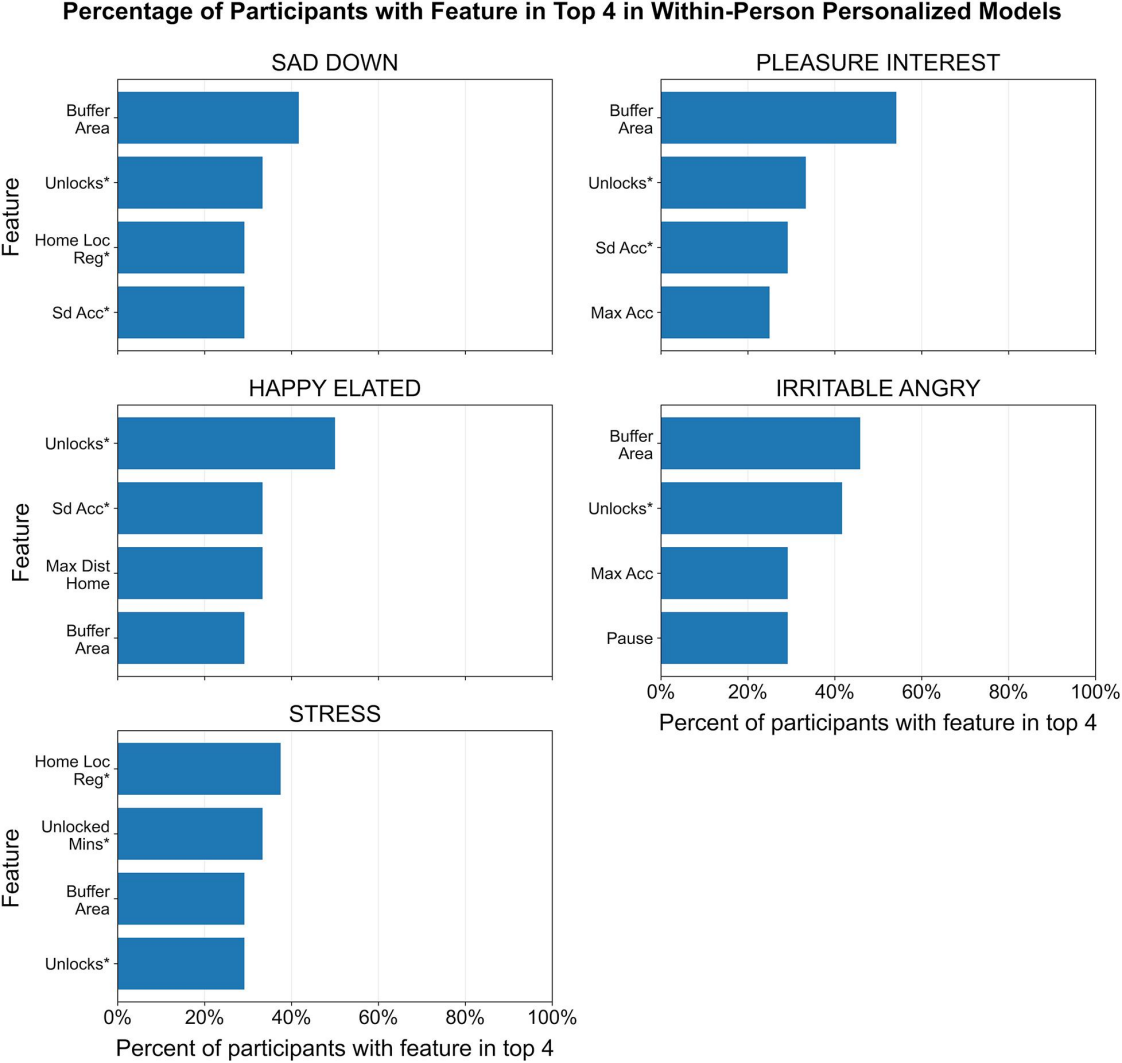
Bar plots of distribution of features most frequently ranked among the top four across participants in model 3 within-person personalized models.

In Model 3 Within-subject Person Personalized models, feature importance and the direction of effects varied across individuals. No behavioral feature was consistently top-ranked. For example, the most frequent predictor of *happy/elated* (phone unlocks) appeared in the top four most important features of only 50% of participants. See Figure 8 for the distribution of features most frequently ranked among the top four by importance across participants. Additionally, permutation importance values varied substantially across individuals. For example, for *irritable/angry*, phone unlocks had a median permutation importance of 0.017 (interquartile range = 0.006–0.055), indicating heterogeneity in how strongly the feature influenced predictions. SHAP analysis further showed inconsistent directions of effect, with mean SHAP values across participants for a given feature near zero. For example, among the top four features for *sad/down*, values ranged from −0.001 to 0.002.

## 4 Discussion

### 4.1 Overview of study and key findings

This study examined the predictive performance of personalized (idiographic) and group (nomothetic) regression models for affect and stress using passive smartphone sensor data. We compared three types of modeling strategies: (1) Model 1 Group General, consisting of group regression models trained across participants, (2) Model 2 Group Personalized, models trained across participants but incorporating participant ID as a feature, and (3) Model 3 Within-Person Personalized, models trained separately on each individual's data. Across five measures of affect and stress, we evaluated model performance using RMSE and R^2^, assessed cross-validation discrepancies, and benchmarked against intercept-only baselines. Our findings show that different model types had complementary strengths and weaknesses. Model 1 Group General models, when evaluated on individuals included in both training and test sets, identified behaviors that were associated with affect or stress across people and outperformed intercept-only comparison (models of average affect/stress across people). However, these associations failed to generalize to new people. Model 2 Group Personalized models capitalized on power by training across the group but modeling person-level behaviors, and offered the most robust compromise between predictive accuracy and stability. However, benchmarking showed that participant ID contributed most of Model 2's predictive power, suggesting that model performance was driven by average between-person differences in affect or stress. Finally, Model 3 Within-Person Personalized models identified behaviors that most strongly predicted outcomes for each person, and achieved lowest error, but model reliability was mixed and should therefore be interpreted with caution. Although personalized modeling approaches (Models 2–3) outperformed general models (Model 1), patterns of results suggest that larger within-person datasets may be needed to identify person-level behaviors that track with affect or stress. Together, results provide insight into modeling considerations that should be accounted for in digital phenotyping research, but results related to specific behaviors that correspond with outcomes should be interpreted with caution.

### 4.2 Modeling comparison

Results showed that models that allowed for personalized prediction (Models 2–3) outperformed models that assumed uniform prediction across people (Model 1) for all outcomes. The strong performance of personalized models aligns with, and extends, prior research suggesting that idiographic modeling may be more effective in capturing intra-individual variability (64). Further, this result is consistent with the emerging shift toward personalized modeling in the digital phenotyping of depression-related symptom dynamics. In a systematic review of digital biomarkers in depression, the authors concluded that personalized modeling is more robust than group-level predictions (17), in line with our findings. In the present study, this pattern may support the idea that the strength and weighting of behavioral predictors of affective outcomes vary across people (59, 62). However, given mixed reliability in the explained variance for Model 3, this interpretation remains an open question.

Within-Person Personalized models (Model 3) achieved the lowest error, which may reflect strong individual fit, however, this result should be interpreted with caution, as dataset-level performance metrics may obscure possible dependencies that can inflate apparent performance. Further, explained variance was less stable at the fold level, with negative values suggesting that performance was worse than simply predicting the mean. This result suggests that Within-Person Personalized may not generalize across time within the same individual.

Group Personalized models (Model 2) offered the best balance of accuracy and stability across evaluation metrics, supporting the utility of modeling approaches that allow for learning both group-level patterns and person-specific deviations. However, in Model 2, participant ID was the most important feature in predicting affect and stress, and inclusion of behavioral features in Model 2 failed to improve performance over and above modeling between-person differences in outcomes. Hence, in Model 2, person-to-person differences in average levels of affect or stress explained the majority of variance in the model. Our sample size precludes stratified modeling by gender, however, we note that gender is a possible contributor to between-person differences captured by participant ID. Prior digital phenotyping research has shown that including gender as a predictor of depression can improve model performance (74, 75). Further, some behavioral features may differ systematically by gender, as suggested by studies showing gender differences in smartphone usage (76–78), as well as differences in sleep patterns measured via wearable devices (79).

Group General models (Model 1) provided mixed evidence that daily behaviors track with affect or stress across people. Group General models outperformed intercept-only models when predicting future outcomes for known individuals, suggesting that these behavioral features may hold some predictive value. However, model performance dropped substantially when predicting outcomes for new individuals, suggesting that these behavioral associations struggled to generalize to new people and were limited in capturing individual variability. This finding highlights a key challenge in developing nomothetic digital biomarkers for mental health: although general models are intended to apply broadly across people, they may fail to do so because they do not capture individual variability.

Notably, participant identity strongly influenced performance across all models in which it was included. However, benchmarking comparisons with the Group General model (feature-only model) showed better performance than intercept-only models. These findings suggest that sensor-based behavioral features may independently track affect and stress without participant identity, but it is noted that features may also capture stable between-person differences even in the absence of including identity as a feature.

Our findings extend on prior research (59, 62, 66) by directly comparing general, hybrid (personalized group), and fully idiographic (within-subject) machine learning models in the context of smartphone-based prediction of affect and stress. Hybrid models, which incorporated subject-level information into general models, performed well, with the best balance of accuracy and stability across performance metrics. However, performance was driven by participant ID, with behavioral features adding little incremental variance. This finding indicates that the model captures stable differences in average affect or stress, rather than consistent associations between behavior and affective outcomes across individuals. Fully idiographic models indicated strong individual fit, but showed unstable performance, limiting confidence about within-person effects. This work emphasizes the importance of model comparisons to distinguish the relative contributions of between-person differences in outcomes vs. within-person fluctuations in (e.g., behavioral) predictors.

### 4.3 Daily behaviors in relation to affect and stress

Importance of behavioral features in predicting affect or stress varied across people and modeling approaches. In Model 1 Group General models, features reflecting activity, mobility, and phone use appeared among the top predictors. For example, Median Acceleration, a feature reflecting physical activity, appeared among the top four most predictive behavioral features for all affect and stress outcomes, consistent with theoretical models linking activation to improved mood (41). However, as noted above, Model 1 showed poor generalizability to new people. In Model 2 Group Personalized models, feature importance analyses showed limited or no predictive utility, over and above participant identity. Finally, in Model 3 Within-Person Personalized models, the importance of specific behavioral features varied widely across individuals and SHAP analyses further revealed inconsistent directions of effect. These patterns point to heterogeneity in the relationships between behavior and affect or stress, consistent with prior research highlighting heterogeneity in behavioral profiles and psychological processes (10, 80–82). Notably, several features included in the present study capture variability-related constructs (e.g., Home Location Regularity, Standard Deviation Acceleration), which have been consistently identified as robust markers in depression-related digital phenotyping research (17), supporting the relevance of the feature set used. However, results regarding behaviors that predict daily affect should be interpreted with caution, given variability in the predictive utility of behaviors across models and limited reliability of within-person models. Despite a relatively large (compared with prior research) within-person dataset and period of data collection, the results of the present study do not provide conclusive evidence of behavioral predictors that predict affect or stress.

### 4.4 Considerations in model evaluation

Evaluating model performance in digital phenotyping presents several methodological challenges and requires careful consideration of modeling goals.

First, performance metrics are influenced by dataset size, with some modeling approaches more vulnerable than others to variability in performance as a function of both number of participants and within-person samples, as cross-validation estimates become increasingly unstable with fewer observations (83). In the present study, Model 3 Within-Person Personalized models yielded the lowest error when measured at the dataset-level (i.e., metrics calculated by aggregating predictions), but the explained variance of these models depended on the approach to cross-validation, suggesting models may overfit moderately-sized within-subject datasets. Performance instability may stem from the smaller number of observations available per person, which results in limited training data within each fold of cross-validation. This finding raises an important consideration about the minimum number of samples required from an individual participant to achieve accurate and reliable predictions. In contrast, Model 2 Group Personalized models, trained across the full dataset but incorporating participant ID, benefited from larger training sets and exhibited more stable performance; however, within-person fluctuations in behavior provided limited explanatory power in Model 2, which may also indicate insufficient within-person samples.

Second, modeling approaches must consider whether the goal is to develop models that generalize across people or within a person over time (29). As Saeb et al. emphasize, when the modeling goal is to predict outcomes for new individuals, cross-validation should be structured such that training and test sets include data from entirely different participants (29). However, if there is no expectation that a model will generalize to new people, but rather that it should generalize to new time periods within the same individual, then standard k-fold cross-validation may be appropriate. Notably, using standard k-fold cross-validation, in which data from the same individual may appear in both training and test splits, can inflate apparent predictive performance across individuals. In the present study, Model 1 Group General models demonstrated poor generalizability when evaluated using grouped k-fold cross-validation, in which participants were held out of the training set. In contrast, performance improved under standard k-fold cross-validation, where individuals appeared in both training and test sets. This suggests that models that link behaviors to affective outcomes may perform reasonably well when predicting future outcomes for known individuals but generalize poorly to new people. Consistent with this interpretation, personalized models outperformed group models, and there was some evidence that different features carried different predictive weight or even exhibited opposite effects depending on the individual. These findings align with prior research demonstrating that personalized models often outperform generalized models in predicting affective outcomes (59, 62, 66), and reflect broader trends in precision mental health that emphasize the need for idiographic modeling frameworks (64).

Third, complex models should outperform simple models in comparative analyses. Most models showed clear improvements over their intercept-only counterparts, indicating that predictors meaningfully contributed to model performance. However, results also emphasize the importance of comparing models against several benchmarks to better understand the variable(s) that drive performance. These comparisons highlight the value of using simple baselines to contextualize predictive gains and evaluate the true added value of feature-based models.

Results from the present model comparisons point to a broader issue in the field: the relative absence of standardized evaluation practices. While many have highlighted the considerable promise of digital phenotyping for mental health research (23, 70, 84), these methods remain relatively new to psychological science and present notable methodological challenges (23, 84, 85). In response to the field's rapid growth, researchers have called for collaborations between psychologists and computer scientists (84–86), as well as the development of shared guidelines, reporting standards, and benchmarks to facilitate more consistent model evaluation (23, 85, 87). Moreover, researchers emphasize the importance of choosing context-sensitive methods that mirror the intended clinical use case and consider the target population (29, 84, 86). Future research should explore approaches that improve robustness and generalizability in models, adopt evaluation strategies that reflect both sample size and modeling intent, and leverage larger within-person datasets to identify person-level behavioral predictors of affective outcomes. We recommend that future studies engage in systematic model comparisons including comparing against benchmark models, report evaluation methods clearly, and match model design to the intended application. Standardized, context-sensitive evaluation will be essential for meaningful progress and real-world impact.

### 4.5 Limitations and future directions

Several limitations should be considered when interpreting these findings. First, the sample consisted of emerging adults, but all participants were college students, and had limited racial, ethnic, and socioeconomic diversity. Although the sample was representative of the local university student body, future research in samples with greater demographic, developmental and clinical diversity is needed to evaluate the generalizability of findings. Second, we used a non-clinical sample. As a result, it remains unclear whether our results would generalize to clinical depression. Future work should test these models in clinical populations to evaluate their translational utility. Third, missing data are an inherent challenge in passive sensing studies (39, 49) and may have affected model performance despite preprocessing efforts (see Supplementary Material). Additional research exploring the impact of user data availability on model performance is warranted. Fourth, our sample size is insufficient to support stratified modeling by gender. Demographic influences, such as gender, may contribute to the between-person differences captured by participant identity in the models, and behavioral features may systematically vary across demographic groups. Future work is recommended to better evaluate whether incorporating gender into models improves their performance. Fifth, analyses were limited to iPhone users, which may have introduced unintended bias and limited the generalizability of the findings. Future work should examine whether users of different smartphone operating systems respond differently to EMA studies, assess platform-related differences in sensor data, and develop and validate platform-agnostic feature extraction methods. Finally, the primary measure of mood was momentary affect, assessed once per day. This brief, momentary assessment design was aimed at fending against participant burden, but also has weaknesses as a measure of depression. For example, it may fail to capture within-day affective dynamics or evaluate time periods of sufficient duration to measure depression. Future research that incorporates more frequent or more extended periods of assessment can provide complementary insight.

## 5 Conclusion

This study compared machine learning models, using general and personalized modeling approaches, for predicting daily affect and stress from passively sensed smartphone data in emerging adults. Model types had complementary strengths and weaknesses; results suggest the importance of accounting for individual variability when predicting affective states but also point to the need for future research using larger within-person datasets to determine person-level behaviors that best predict affective and stress outcomes. Advancing personalized and hybrid modeling strategies will be essential for such future research, and to explore translational applications.

## Data Availability

The datasets presented in this article are not readily available because the datasets analyzed in this study are not publicly available to protect participant privacy. Participants did not consent to data sharing and some of the data types are identifying. Requests to access the datasets should be directed to Roselinde H. Kaiser, Roselinde.Kaiser@colorado.edu.
