# Supplementary material for: Digital phenotyping of affect and stress in emerging adults
Source: Front Digit Health. 2026 Jun 4;8:1799541. doi: 10.3389/fdgth.2026.1799541 (PMC13275722; doi:10.3389/fdgth.2026.1799541)
Supplement: Supplementary file 1 [file Datasheet1.docx]

Supplementary Material

# Impact of Data Availability

To examine the impact of user data availability on model predictions, we conducted Spearman correlation analyses between the amount of user data and mean predicted affective or stress EMA scores. As shown in Figure S1, there were no significant correlations between user data availability and the model’s predicted mean EMA scores for any of the affective outcomes. Spearman correlation coefficients (ρ) were generally close to zero, and all p-values were far from significance (p > 0.5). For example, the correlation for *sad/down* was ρ = -0.006 (p = 0.979), and for *happy/elated* it was ρ = 0.002 (p = 0.992). These results suggest that the amount of data a participant contributed did not systematically influence the model’s average predictions.

To examine the impact of user data availability on model performance, we conducted Spearman correlation analyses between the amount of user data and RMSE and R² model performance metrics measured by fold and by dataset. To account for multiple tests per EMA, p-values were adjusted using the Benjamini-Hochberg false discovery rate (FDR) correction. Correlations between user data availability and model performance are presented in Table S1. Correlations were weak and non-significant across RMSE metrics. For R² metrics, somewhat higher effect sizes were observed but no associations survived FDR correction. Additional research exploring the impact of user data availability on model performance is warranted.

To assess whether EMA missingness was associated with passively sensed behavioral patterns, we fit a series of generalized linear mixed models for each sensor derived behavioral feature (z-score normalized), with participant ID as a random intercept. To account for multiple comparisons across features, p-values were adjusted using the Benjamini-Hochberg FDR correction. Across behavioral features, higher phone use was associated with lower odds of EMA missingness. Odds ratios of features with significant associations are presented in Figure S2.


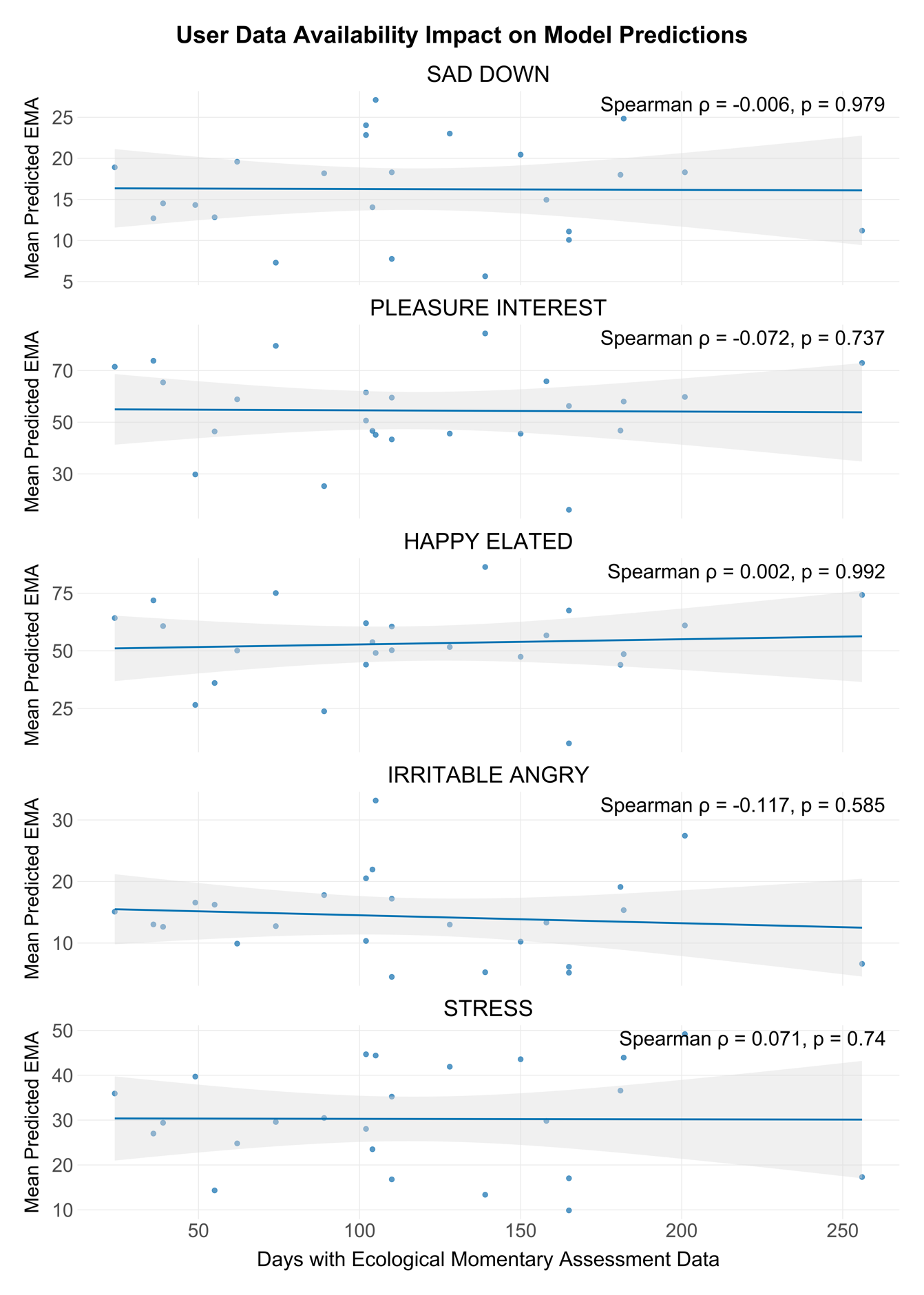


**Supplementary Figure 1.** Impact of User Data Availability on Model Predictions

**Supplementary Table 1.** Impact of User Data Availability on Model Performance

| EMA | Metric | Spearman ρ | Uncorrected p-value | FDR corrected p-value |
| --- | --- | --- | --- | --- |
| SAD DOWN | RMSE (Dataset) | 0.147 | 0.493 | 0.493 |
|  | RMSE (Fold) | 0.22 | 0.302 | 0.403 |
|  | R² (Dataset) | 0.231 | 0.277 | 0.403 |
|  | R² (Fold) | 0.417 | 0.043* | 0.17 |
| PLEASURE INTEREST | RMSE (Dataset) | -0.023 | 0.915 | 0.915 |
|  | RMSE (Fold) | -0.113 | 0.600 | 0.346 |
|  | R² (Dataset) | 0.288 | 0.173 | 0.8 |
|  | R² (Fold) | 0.417 | 0.043* | 0.17 |
| HAPPY ELATED | RMSE (Dataset) | -0.185 | 0.386 | 0.386 |
|  | RMSE (Fold) | -0.191 | 0.370 | 0.386 |
|  | R² (Dataset) | 0.266 | 0.209 | 0.386 |
|  | R² (Fold) | 0.388 | 0.061 | 0.245 |
| IRRITABLE ANGRY | RMSE (Dataset) | -0.07 | 0.744 | 0.867 |
|  | RMSE (Fold) | -0.036 | 0.867 | 0.703 |
|  | R² (Dataset) | 0.199 | 0.352 | 0.867 |
|  | R² (Fold) | 0.323 | 0.124 | 0.496 |
| STRESS | RMSE (Dataset) | -0.156 | 0.466 | 0.692 |
|  | RMSE (Fold) | -0.085 | 0.692 | 0.692 |
|  | R² (Dataset) | -0.103 | 0.633 | 0.692 |
|  | R² (Fold) | 0.087 | 0.685 | 0.692 |

Abbreviations: EMA, ecological momentary assessment; RMSE, Root Mean Square Error


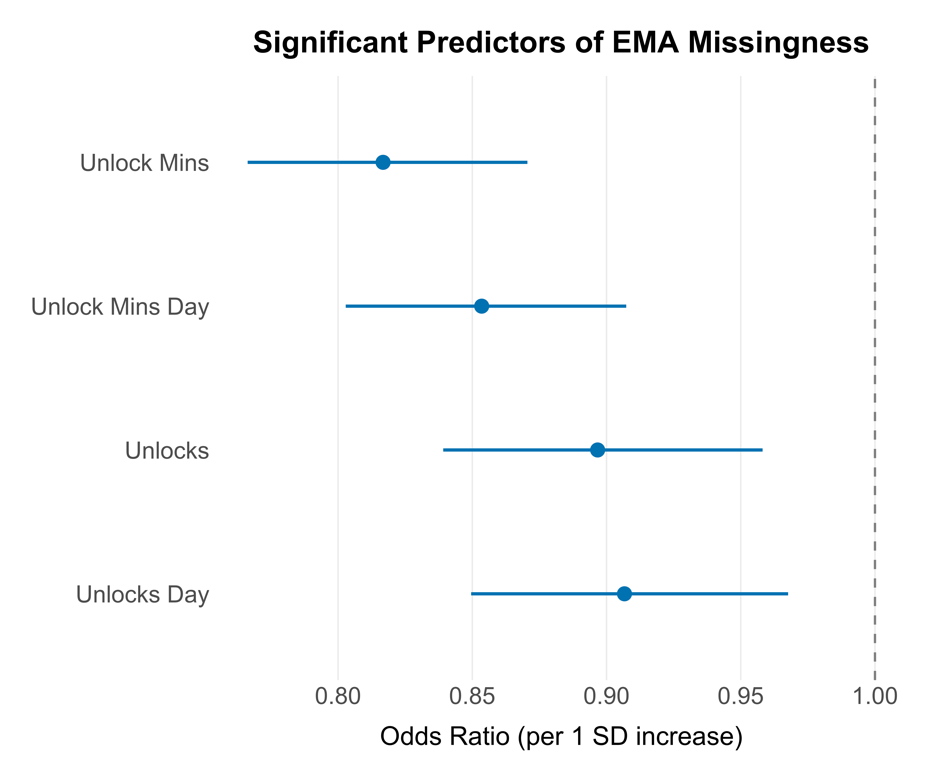


**Supplementary Figure 2.** Significant predictors of EMA presence

# Identifying Subgroups with K-Means Clustering

To explore data-driven subgroups of emerging adults with distinctive profiles of depression risk, we conducted a k-means clustering analysis on the Model 2 Group Personalized model results for *sad/down*. We identified the four features with the highest mean absolute SHAP value for the sad/down Model 2. Results showed that across the group, the four features with highest average importance were Maximum Distance From Home, Phone Unlocks composite, Median Magnitude Acceleration composite, and Standard Deviation Magnitude Acceleration composite. We then used participant-specific mean SHAP values for these features to perform k-means clustering (k = 2,3, and 4). Figure S3 displays the resulting cluster profiles in heatmaps. This method may offer a means for identifying subgroups of emerging adults with different risk profiles, an important strategy for understanding heterogeneity in depression. We recommend further research exploring data-driven subgroup identification in larger datasets, using both Model 2 and Model 3 approaches for distinguishing important features.


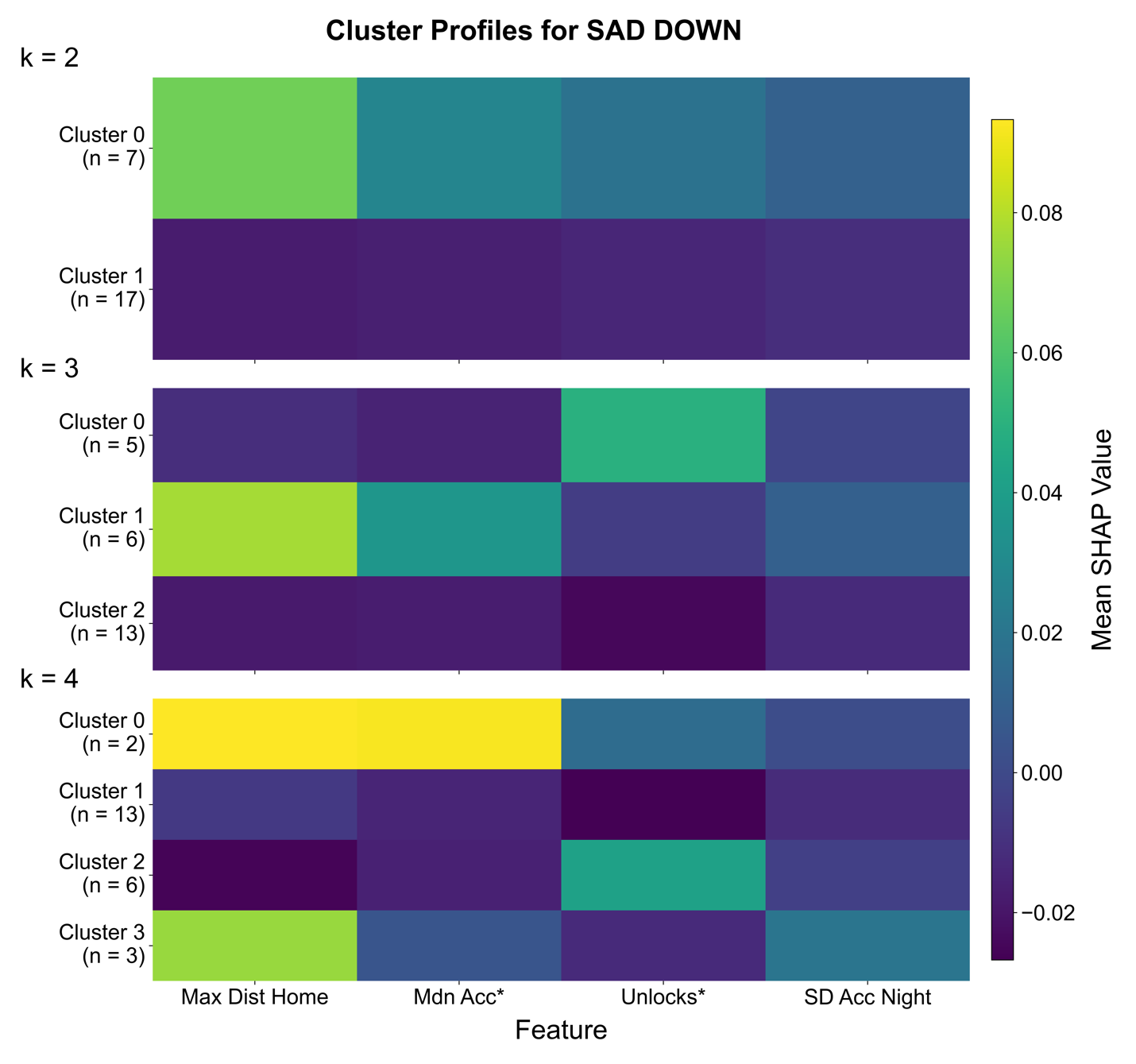


**Supplementary Figure 3:** Heatmap of cluster profiles identified in K = 2,3,4 k-means clustering analysis on the Group Personalized model for sad/down

# XGBoost Classifier

Personalized and general model classifier analyses were performed. For XGBoost classifier models, EMA outcomes were classified into three categories (low, medium, high) using a tertile split on scores normalized within-person. Under this scheme, a balanced accuracy or weighted F1-score near 0.33 reflects chance-level performance. Model 1 Group General models yielded weighted F1-scores ranging from 0.420 (*sad/down*) to 0.384 (*pleasure/interest*), and balanced accuracy of 0.411 (*sad/down*) to 0.384 (*pleasure/interest*). Model 2 Group Personalized models, which were trained across the group but included participant ID as a feature, yielded weighted F1-scores ranging from 0.424 (*sad/down*) to 0.380 (*pleasure/interest*), and balanced accuracy of 0.415 (*sad/down*) to 0.379 (*pleasure/interest*). Model 3 Within-Person Personalized, trained within-subject, yielded weighted F1-scores ranging from 0.490 (*sad/down*) to 0.456 (Pleasure/Interest and *Stress*), and balanced accuracy of 0.481 (*sad/down*) to 0.453 (*stress*). However, fold-level metrics, calculated by averaging performance across cross-validation folds, revealed some discrepancies. For Model 3 Within-Person Personalized models, fold-level balanced accuracy ranged from 0.375 (*pleasure/interest*) to 0.402 (*sad/down*), substantially lower than their dataset-level equivalents. This discrepancy suggests that within-person sample sizes may have been too small for stable validation at the fold level, similar to patterns observed in the regression models. For Models 1-2, fold-level metrics were slightly lower than dataset-level values, with minor deviations (e.g., fold-level balanced accuracy for *pleasure/interest* was 0.366 in both models, compared to 0.384 and 0.379, respectively, at the dataset level). See Figure S4 for a summary of model performance.

At the dataset level, Model 3 Within-Person Personalized models yielded the highest weighted F1-scores across EMAs. However, when focusing on fold-level performance, differences between approaches were modest. Model 3 continued to show some advantage in weighted F1-scores, suggesting greater overall classification accuracy, but gains were relatively small, and no approach clearly and consistently outperformed across both metrics and constructs. These results stand in contrast to the clearer separation in model performance observed for regression models. In this context, while Model 3 within-subject personalization showed some benefit for overall classification accuracy, improvements were modest, and no modeling approach emerged as a consistent best performer across all outcomes.

# Additional Tables and Figures

**Supplementary Table 2.** Full set of behavioral features derived from digital sensor data

| Feature | Description | Units | Sensor | Source | Category |
| --- | --- | --- | --- | --- | --- |
| Distance | Total distance traveled | miles | GPS | In house | Mobility |
| Span | Furthest distance between two points | miles | GPS | In house | Mobility |
| Buffer area | Activity space – buffer space | sq meters | GPS | In house | Mobility |
| Convex Area | Activity space – convex hull | sq meters | GPS | In house | Mobility |
| Max Acc | Maximum magnitude of acceleration | *(m/s^2^)* | Accelerometer | In house | Movement |
| Max Acc Day | Maximum magnitude of acceleration during day hours (8am – 4pm) | *(m/s^2^)* | Accelerometer | In house | Movement |
| Max Acc Night | Maximum magnitude of acceleration during night hours (10pm – 4am) | *(m/s^2^)* | Accelerometer | In house | Movement |
| Median Acc | Median magnitude of acceleration | *(m/s^2^)* | Accelerometer | In house | Movement |
| Median Acc Day | Median magnitude of acceleration during day hours (8am – 4pm) | *(m/s^2^)* | Accelerometer | In house | Movement |
| Median Acc Night | Median magnitude of acceleration during night hours (10pm – 4am) | *(m/s^2^)* | Accelerometer | In house | Movement |
| SD Acc | Standard deviation magnitude of acceleration | *(m/s^2^)* | Accelerometer | In house | Movement |
| SD Acc Day | Standard deviation magnitude of acceleration during day hours (8am – 4pm) | *(m/s^2^)* | Accelerometer | In house | Movement |
| SD Acc Night | Standard deviation magnitude of acceleration during night hours (10pm – 4am) | *(m/s^2^)* | Accelerometer | In house | Movement |
| Unlocks | Number of screen unlocks |  | Phone log | In house | Phone Use |
| Unlocks Day | Number of screen unlocks during day hours (8am – 4pm) |  | Phone log | In house | Phone Use |
| Unlocks Night | Number of screen unlocks during night hours (10pm – 4am) |  | Phone log | In house | Phone Use |
| Unlock Mins | Number of minutes the screen is unlocked | minutes | Phone log | In house | Phone Use |
| Unlock Mins Day | Number of minutes the screen is unlocked during day hours (8am – 4pm) | minutes | Phone log | In house | Phone Use |
| Unlock Mins Night | Number of minutes the screen is unlocked during night hours (10pm – 4am) | minutes | Phone log | In house | Phone Use |
| Home Time | Time spent at home. Home is the most visited significant location between 8 pm and 8 am | minutes | GPS | RAPIDS^a^ | Mobility |
| Dist Travelled | Total distance traveled over a day | miles | GPS | RAPIDS | Mobility |
| Rog | Radius of gyration – A measure in meters of the area covered by a person over a day | meters | GPS | RAPIDS | Mobility |
| Max Diam | Maximum diameter is the largest distance between any two pauses | meters | GPS | RAPIDS | Mobility |
| Max Home Dist | Maximum distance from home | meters | GPS | RAPIDS | Mobility |
| Sig Locs Visited | Number of significant locations visited during the day. Significant locations are computed using k-means clustering. | locations | GPS | RAPIDS | Exploration |
| Avg Flight Len | Mean length of all flights. | meters | GPS | RAPIDS | Mobility |
| SD Flight Len | Standard deviation of the length of all flights. | meters | GPS | RAPIDS | Mobility |
| Avg Flight Dur | Mean duration of all flights |  | GPS | RAPIDS | Mobility |
| SD Flight Dur | Standard deviation of the duration of all flights. |  | GPS | RAPIDS | Mobility |
| Prob Pause | The fraction of a day spent in a pause (as opposed to a flight) |  | GPS | RAPIDS | Mobility |
| Sig Loc Entropy | Significant location entropy (Shannon’s entropy) |  | GPS | RAPIDS | Exploration |
| Circdn Rtn | circadian routine that can take any value between 0 and 1 |  | GPS | RAPIDS | Circadian Routine |
| Wkend Day Rtn | Same as Circdn Rtn but computed separately for weekends and weekdays. |  | GPS | RAPIDS | Circadian Routine |

^a^RAPIDS refers to the Reproducible Analysis Pipeline for Data Streams pipeline (1), an open-source platform that supports feature extraction from data collected via mobile devices.

**Supplementary Table 3.** Hyperparameter values tested in tuning across models

| Hyperparameter | Values Tested |
| --- | --- |
| Number of trees | 30, 50, 100, 150, 250 |
| Maximum tree depth | 3, 5, 7, 10, 13 |
| Learning rate | 0.01, 0.1, 0.2 |
| Subsample ratio | 0.7, 0.8, 1.0 |

**Supplementary Table 4.** Cross-validation: number of folds and dataset size in each fold

| Model | K-splits | Train size per fold | Test size per fold |
| --- | --- | --- | --- |
| Group General | 5 | 2228 | 558 |
| Group Personalized | 5 | 2228 | 558 |
| Within-Person Personalized^a^ | 2 | 18 | 18 |
|  |  | 37 | 37 |
|  |  | 128 | 128 |
|  |  | 44 | 45 |
|  |  | 100 | 101 |
|  |  | 52 | 53 |
|  |  | 79 | 79 |
|  |  | 31 | 31 |
|  |  | 51 | 51 |
|  |  | 19 | 20 |
|  |  | 69 | 70 |
|  |  | 82 | 83 |
|  |  | 27 | 28 |
|  |  | 52 | 52 |
|  |  | 75 | 75 |
|  |  | 12 | 12 |
|  |  | 91 | 91 |
|  |  | 51 | 51 |
|  |  | 55 | 55 |
|  |  | 64 | 64 |
|  |  | 24 | 25 |
|  |  | 82 | 83 |
|  |  | 55 | 55 |
|  |  | 90 | 91 |

^a^For Personalized models, rows under *Train size per fold* and *Test size per fold* correspond to participants.

**Supplementary Table 5.** XGBoost Regression Performance Metrics (Mean Across Folds)

| Model | EMA | RMSE (SE)^a^ | MSE (SE) | MAE (SE) | R² (SE) | Pearson’s r |
| --- | --- | --- | --- | --- | --- | --- |
| Group General | Sad Down | 17.016 (0.438) | 289.557 (14.895) | 11.974 (0.191) | 0.138 (0.006) | 0.375 |
|  | Pleasure Interest | 22.385 (0.170) | 501.102 (7.630) | 18.073 (0.134) | 0.355 (0.005) | 0.611 |
|  | Happy Elated | 22.675 (0.333) | 514.144 (15.118) | 18.168 (0.301) | 0.376 (0.012) | 0.626 |
|  | Irritable Angry | 16.765 (0.349) | 281.063 (11.705) | 11.235 (0.127) | 0.197 (0.008) | 0.447 |
|  | Stress | 22.421 (0.216) | 502.714 (9.705) | 17.301 (0.159) | 0.213 (0.011) | 0.470 |
| Group Personalized | Sad Down | 16.622 (0.406) | 276.293 (13.497) | 11.530 (0.163) | 0.177 (0.008) | 0.429 |
|  | Pleasure Interest | 20.040 (0.159) | 401.587 (6.354) | 15.478 (0.118) | 0.483 (0.007) | 0.693 |
|  | Happy Elated | 20.291 (0.323) | 411.707 (13.094) | 15.520 (0.227) | 0.500 (0.011) | 0.703 |
|  | Irritable Angry | 16.096 (0.418) | 259.094 (13.455) | 10.643 (0.205) | 0.259 (0.019) | 0.509 |
|  | Stress | 20.722 (0.320) | 429.396 (13.242) | 15.793 (0.227) | 0.328 (0.013) | 0.568 |
| Within-Person Personalized | Sad Down | 15.041 | 226.233 | 10.996 | -0.080 | 0.466 |
|  | Pleasure Interest | 18.415 | 339.106 | 14.713 | -0.061 | 0.732 |
|  | Happy Elated | 18.795 | 353.243 | 15.022 | -0.094 | 0.718 |
|  | Irritable Angry | 13.535 | 183.198 | 10.204 | -0.163 | 0.568 |
|  | Stress | 20.095 | 403.799 | 16.017 | -0.094 | 0.606 |

^a^Standard error is not included in Model 3 Within-Person Personalized models as metrics were computed from 2-fold cross validation for those models due to smaller sample size in individual models. We implemented 5-fold cross-validation for Models 2-3 and include standard error. See Table S4 for more information on cross-validation folds for each model type. Abbreviations: RMSE, root mean square error; MSE, mean square error; MAE, mean absolute error; SE, standard error; Acc, acceleration; EMA, ecological momentary assessment; Pearson’s r, Pearson correlation between predicted and actual values

**
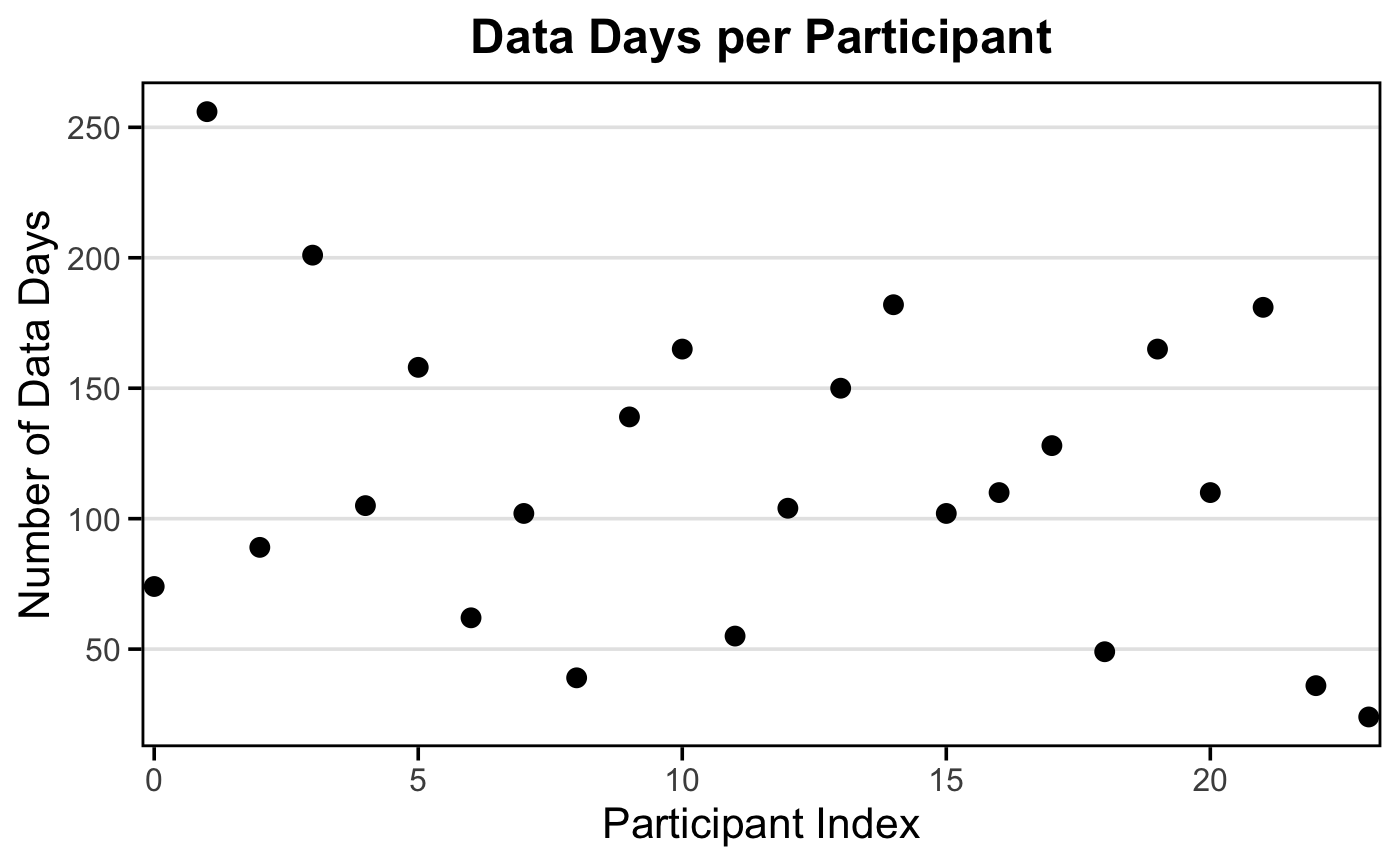
**

**Supplementary Figure 5:** Dot plot showing range of observations per subject.

**
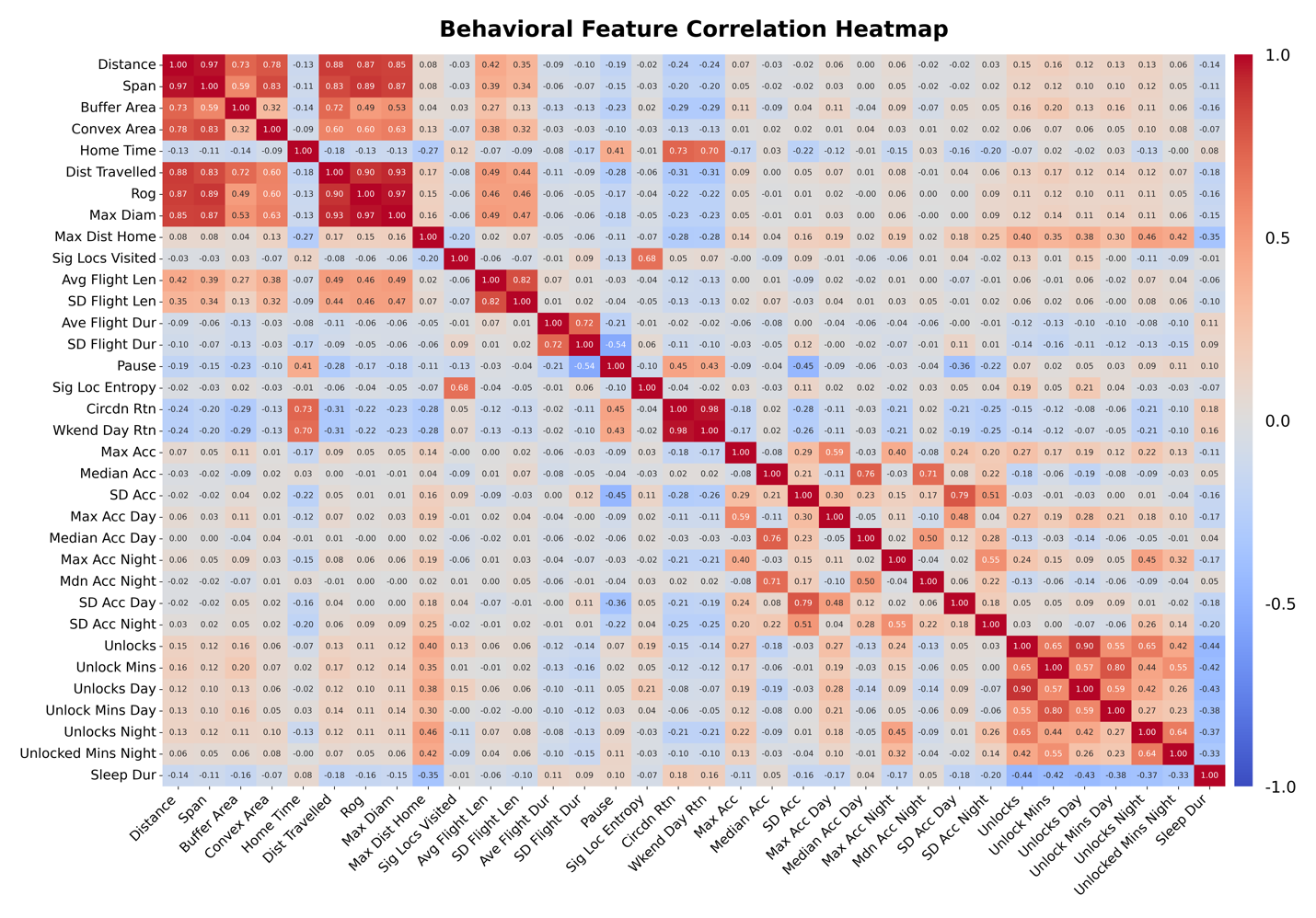
**

**Supplementary Figure 6:** Heatmap matrix of behavioral feature pairwise correlations.

**
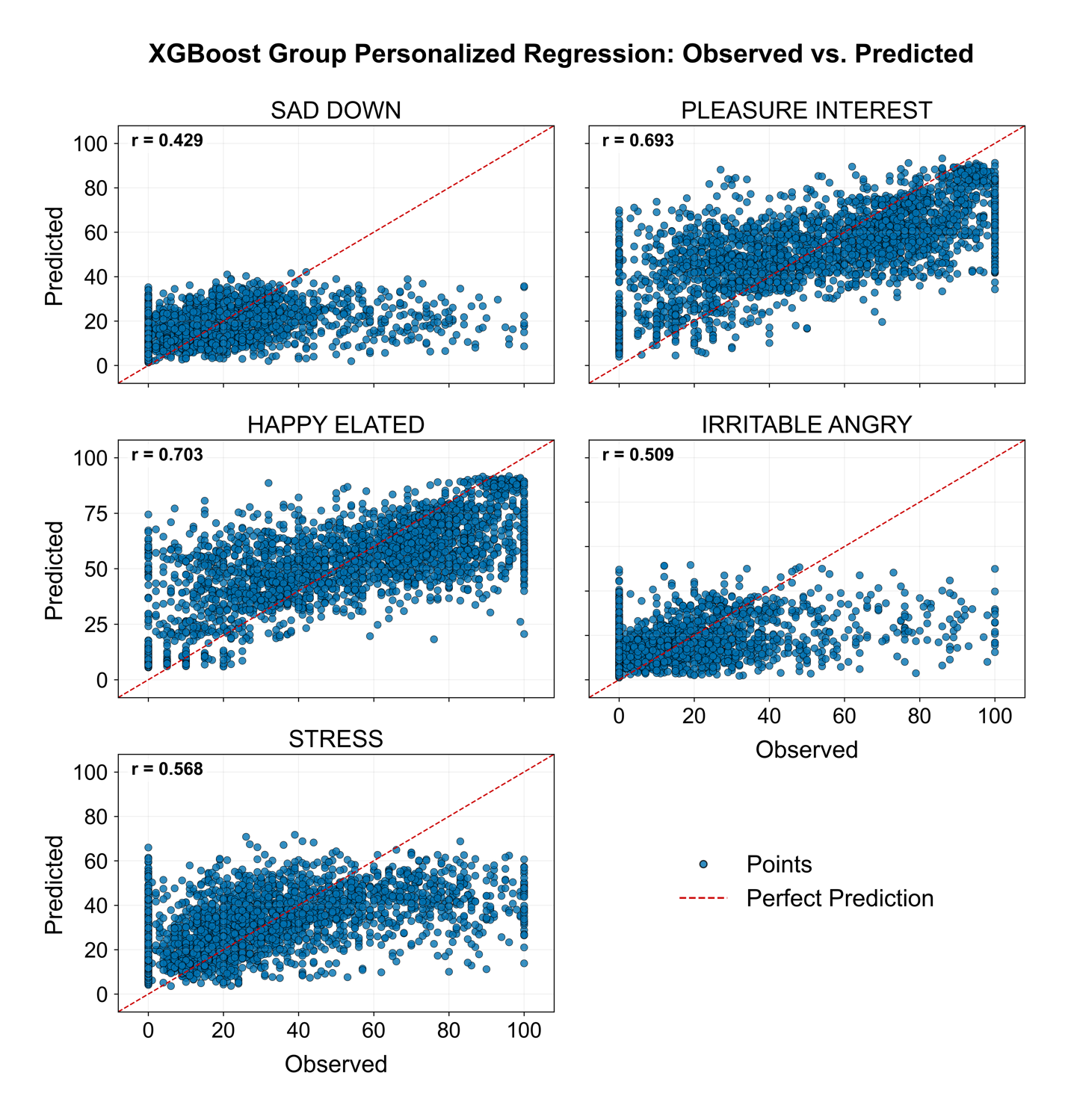
Supplementary Figure 7:** Predicted vs. observed EMA scores for Group Personalized models across all outcomes

# References

1. Vega J, Li M, Aguillera K, Goel N, Joshi E, Khandekar K, et al. Reproducible Analysis Pipeline for Data Streams: Open-Source Software to Process Data Collected With Mobile Devices. Frontiers in Digital Health [Internet]. 2021;Volume 3-2021. Available from: https://www.frontiersin.org/journals/digital-health/articles/10.3389/fdgth.2021.769823
